# Cs_2_Fe_2_(MoO_4_)_3_—A Strongly Frustrated Magnet with Orbital Degrees of Freedom and Magnetocaloric Properties

**DOI:** 10.1021/acs.chemmater.4c01262

**Published:** 2024-07-02

**Authors:** Lenka Kubíčková, Anna Katharina Weber, Martin Panthöfer, Stuart Calder, Angela Möller

**Affiliations:** † Department of Chemistry, Johannes Gutenberg University Mainz, Duesbergweg 10-14, 55128 Mainz, Germany; ‡ Neutron Scattering Devision, Oak Ridge National Laboratory, Oak Ridge, Tennessee 37831, United States

## Abstract

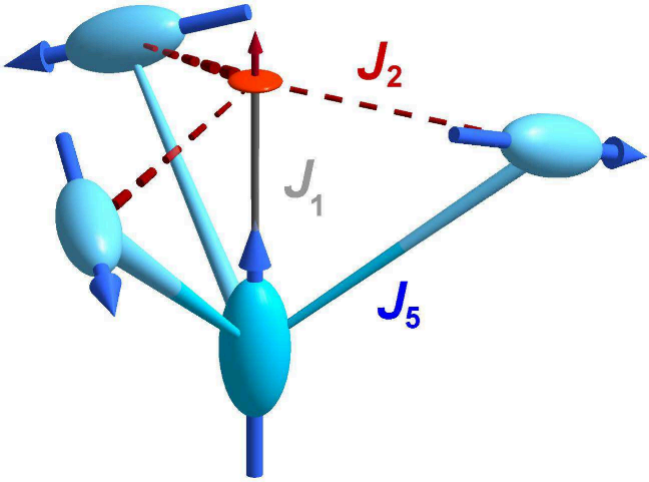

We report an in-depth study of the thermodynamic and magnetocaloric properties of a strongly frustrated magnet, Cs_2_Fe_2_(MoO_4_)_3_. The underlying structure belongs to the double trillium lattice, which consists of two Fe^II^ (*S* = 2) sites with easy-axis and easy-plane single-ion anisotropy. Detailed ^57^Fe Mössbauer spectroscopic investigations along with ligand-field calculations support the existence of disparate ground states. The antiferromagnetic ordered structure is presented by the propagation vector ***k*** = (0,0,0) with noncollinear magnetic moments of 2.97 μ_B_ (Fe1) and 0.17 μ_B_ (Fe2), respectively. Strong and disordered magnetic correlations exist in the temperature regime between *T*_N_ ≈ 1.0 K and |θ_CW_| ≈ 22 K. The large degeneracy of the ground state is investigated in terms of its magnetocaloric response. Magnetization and specific heat measurements indicate a significant magnetocaloric cooling efficiency, making this rare-earth-free compound a promising candidate for cryogenic magnetic refrigeration applications, with refrigeration capacity of 79 J kg^–1^ for Δ(μ_0_*H*) = 8 T.

## Introduction

Magnetic refrigeration, which uses applied magnetic fields to cool matter, is expected to play an important role in improving the energy efficiency of cooling systems. It bases on the magnetocaloric (MC) effect of a magnetic material which, when exposed to a variable magnetic field, alters its temperature as a response to changes in magnetic entropy. Hydrogen liquefaction, which is in high demand and in the focus of the ongoing energy transformation, is one key application.^1−3^ Additionally, magnetic refrigeration offers a cost-effective ^3^He-free alternative to ^3^He–^4^He dilution refrigeration in achieving temperatures below 1 K, which are essential for a rapidly growing range of applications in quantum technologies.^4^

Classical high-temperature MC materials, such as La(Fe_1–*x*_Si_*x*_)_13_,^5^ Gd_5_(Ge_4–*x*_Si_*x*_),^6^ or Fe–Rh^7^ alloys, have been investigated in a narrow temperature range close to their magnetic phase transition. Their cooling efficiency^8^ and performance are often limited due to first-order phase transitions resulting in irreversible losses during thermal and magnetic cycling.^9^ Recent thrusts for enhancing cooling performance at cryogenic temperatures feature rare-earth element materials exhibiting magnetic second-order phase transitions.^10−12^ An attractive alternative posed is frustrated magnets, with their large number of low-lying energy states and inherent high magnetic entropy released over an extended temperature range, especially around 20 K—the boiling point of H_2_—and below.^13−15^ From a materials perspective, a further challenge for implementing MC materials into cooling systems is to ensure isotropic heat transport. Thus, cubic crystal structures are preferred.

Fascinating magnetic ground states are established in the noncentrosymmetric cubic space group *P*2_1_3 and are represented by the famous skyrmion lattices such as the intermetallic phases of the FeSi-type^16,17^ or the antiferromagnetic insulator Cu_2_OSeO_3_.^18,19^ The peculiarity of this space group is that it supports strong geometrical frustration embodied by the trillium lattice,^20^ which presents a three-dimensional network of equilateral corner-sharing triangles formed by magnetic ions. This lattice type is relatively unexplored and magnetic insulators with the trillium motif, such as the MC material Na[Mn(HCOO)_3_] (*S* = 5/2),^21^ are particularly intriguing. The more complex langbeinite type of compounds combines two interconnected trillium lattices, with prominent examples K_2_Ni_2_(SO_4_)_3_ (*S* = 1), a field-induced quantum-spin-liquid candidate^22^ with confirmed continuous spin excitations^23^ or KSrFe_2_(PO_4_)_3_ (*S* = 5/2) with a suggested spin-liquid state.^24^

The classical Heisenberg model of the frustrated trillium lattice with antiferromagnetic interactions predicts a magnetically ordered state^20,25^ with a transition temperature much lower than the Curie–Weiss temperature, *T*_N_ ≪ |θ_CW_|. Within the wide temperature range between these characteristic temperatures, a cooperative paramagnetic state persists. Intriguing avenues that go beyond the simple Heisenberg exchange comprise the introduction of spin–orbit coupling, single-ion anisotropy, antisymmetric exchange, or orbital degrees of freedom to the topology of interconnected trillium lattices.

## Results and Discussion

Microcrystalline Cs_2_Fe_2_(MoO_4_)_3_ was synthesized by the solid-state reaction as described in the Materials and Methods section. Further details on refinements of diffraction data, general methods, and physical properties are provided in the Supporting Information (SI).

### Crystal Structure

The langbeinite type of compounds have the general formula *A*_2_*M*_2_(*T*O_4_)_3_, where *A* represents alkali or alkali-earth metals, *M* transition metals, and *T*O_4_ rigid bridging complex oxides (e.g., SO_4_^2–^ or PO_4_^3–^). Most of these compounds contain *Pearson* hard ions and undergo phase transitions upon cooling.^26−31^ Following the idea that more polarizable, i.e., *Pearson*, softer, larger ions (Cs^+^ and MoO_4_^2–^) are suitable to retain the intriguing noncentrosymmetric structure (space group *P*2_1_3); we choose Cs_2_Fe_2_(MoO_4_)_3_, for which the room temperature structure was reported earlier.^32^

**Figure 1:**
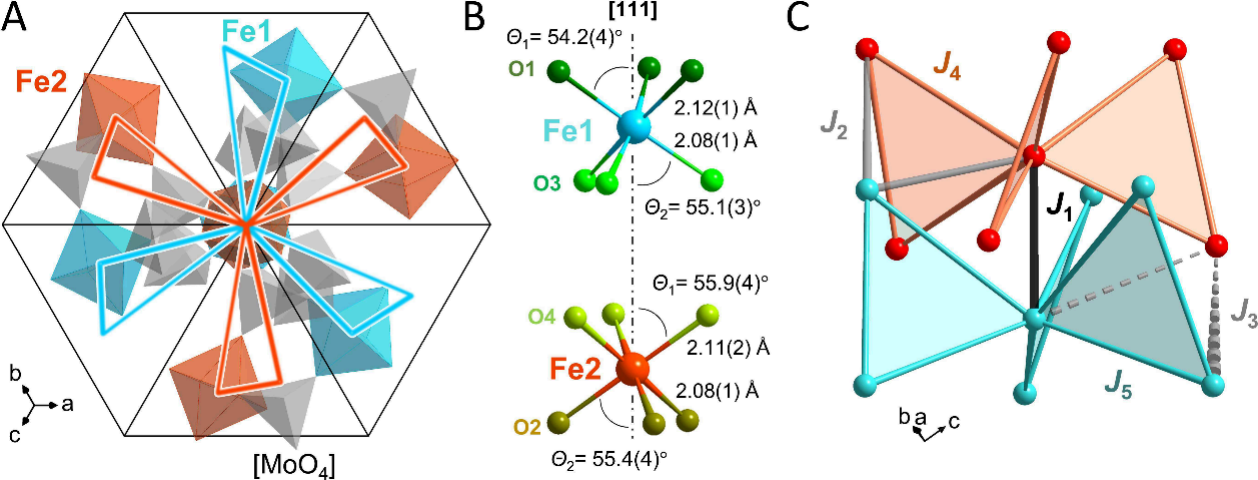
(A) Crystal structure of Cs_2_Fe_2_(MoO_4_)_3_. The double-trillium motif is overlaid (blue: Fe1, red: Fe2), pending edges of the sloped equilateral triangles reach out to neighboring unit cells, and Cs atoms are not shown. (B) [FeO_6_] polyhedra with angles and distances derived from room temperature data. (C) Network of inter- (black/gray) and intratrillium (red/blue) Fe^II^–O^–II^–Mo^VI^–O^–II^–Fe^II^ supersuperexchange pathways, as described in Table 1.

The crystal structure contains two crystallographically nonequivalent Fe^II^ ions (*S* = 2). These are connected solely via [MoO_4_] tetrahedra; see Figure 1A. Each [FeO_6_] polyhedron (*C*_3*v*_ symmetry) contains Fe^II^ ions which are shifted off-center along a 3-fold axis ⟨111⟩ of the cubic unit cell; see Figure 1B. The average angles between the 3-fold axis and Fe–O, Θ_avg_, are 54.65° and 55.65° for Fe1 and Fe2, respectively, whereas Θ = 54.74° corresponds to a regular octahedron. We provide further insights into the electronic structure of the individual iron ions in the subsection on ligand-field calculations and SI.

Figure 1C illustrates the individual trillium lattices of Fe1 (blue) and Fe2 (red). Note the distinct sizes of equilateral triangles of the two sublattices; see Table 1 for the individual Fe1–Fe1 and Fe2–Fe2 distances. The two trillium lattices are interlinked via shorter Fe1–Fe2 distances. These fundamental structural motifs define the principal magnetic interaction pathways, which we now explore further. [MoO_4_] tetrahedra act as linkers between magnetic ions, efficiently mediating the Fe^II^–O^–II^–Mo^VI^–O^–II^–Fe^II^ supersuperexchange (SSE) interaction via the extended 4d-orbitals of Mo. Thereby, each Fe^II^ interacts with six Fe^II^ of the same sublattice and seven Fe^II^ of the other. An overview of these SSE pathways is given in Table 1 and in Figure 1 C.

**Table 1:** Super-superexchange Interaction Pathways for Cs_2_Fe_2_(MoO_4_)_3_, Based on the Room-Temperature Crystal Structure

Inter.	Sites	Distance (Å)	Multiplicity	∠(Fe–Mo–Fe)
*J*_1_	Fe1–Fe2	5.201(5)	1	90.86(9)°
*J*_2_	Fe1–Fe2	5.514(5)	3	98.64(9)°
*J*_3_	Fe1–Fe2	6.509(5)	3	117.52(9)°
*J*_4_	Fe2–Fe2	6.693(5)	6	130.29(9)°
*J*_5_	Fe1–Fe1	6.782(5)	6	129.27(9)°

The magnetic nearest-neighbor (*nn*) interaction represents the intertrillium connectivity. These *nn*-dimers with ∠(Fe1–Mo–Fe2) ≈ 90° are oriented along all 3-fold symmetry axes of the cubic unit cell. The Heisenberg exchange is then drastically reduced, as indicated also by the corresponding very small *J*_1_ parameter for K_2_Ni_2_(SO_4_)_3_.^22,23^ Following these symmetry arguments, the presence of antisymmetric exchange (Dzyaloshinskii–Moriya interaction, DMI^33,34^) introduces a ferromagnetic component to the *nn*-exchange. In contrast, all further inter- and intratrillium lattice interactions *J*_2_–*J*_5_ correspond to antiferromagnetic Heisenberg exchange of next-nearest neighbors (*nnn*). Note that *J*_4_ and *J*_5_ represent the individual trillium lattices in Cs_2_Fe_2_(MoO_4_)_3_, while in K_2_Ni_2_(SO_4_)_3_ these correspond to *J*_3_ and *J*_5_, respectively. This indicates a dependence on the anion size.

### Ligand Field Calculations

**Figure 2:**
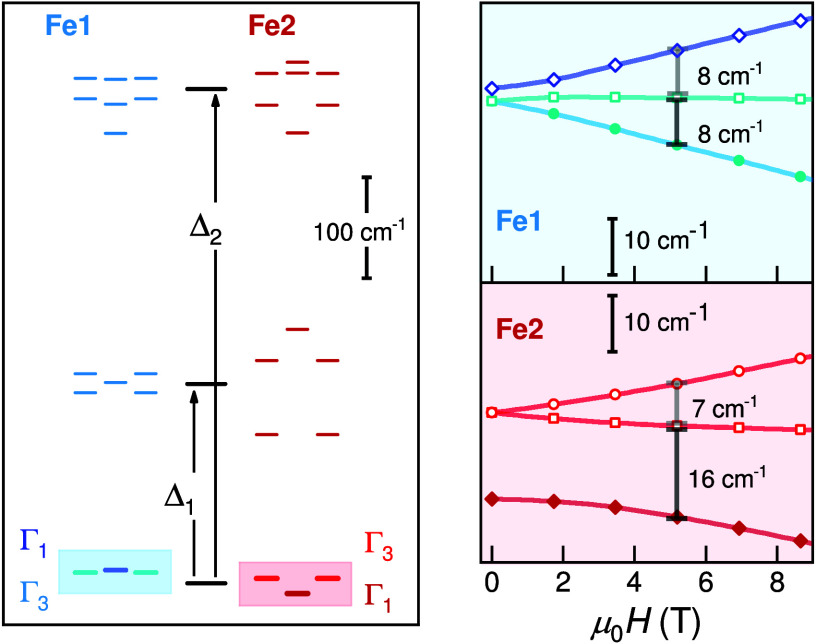
*Left*: Calculated energy levels resulting from spin–orbit coupling for Fe1 and Fe2 in ligand fields with *C*_3*v*_ symmetry, arising from the respective symmetry reduction of the ^5^*T*_2*g*_ state (*O*_*h*_, [FeO_6_]). The averaged energy splitting denoted Δ_1,2_ (black) refers to a simplified three level model; see text. *Right*: The colored boxes show the calculated Zeeman splitting of the lowest energy levels of Fe1 and Fe2 in applied magnetic fields. The relative energy scales are given by bars.

In order to gain insights into the electronic ground state of the individual Fe-sites, we employ the angular overlap model (AOM). The AOM provides a chemically intuitive, semiquantitative parametrization of the ligand-field theory.^35−37^ We performed AOM calculations for the isolated [FeO_6_] polyhedra based on the room-temperature structure (see Figure 1B) in the paramagnetic regime. Details and parameters of the ligand field calculations are provided in SI and Materials and Methods. The trigonally distorted ligand field with *C*_3*v*_ symmetry splits the orbitally degenerate ^5^*T*_2*g*_ ground state of a regular octahedral field with *O*_*h*_ symmetry into a doublet ^5^*E* (the ground state for the Fe1 site) and a singlet ^5^*A*_1_ (the ground state for the Fe2 site). These correspond to an elongation (Fe1) and compression (Fe2) of the respective [FeO_6_] octahedra along the 3-fold axis, and depend on Θ; see Figure 1 B. In Figure 2 (*left*) we show the energy levels arising from spin–orbit splitting for Fe1 and Fe2 in their respective ligand fields of *C*_3*v*_ symmetry. The irreducible representations in *Bethe* notation in zero field are Γ_3_ (Fe1) and Γ_1_ (Fe2) for the lowest energies and are 2-fold and nondegenerate, respectively. It follows that the ground state is magnetic for Fe1 and nonmagnetic for Fe2. The calculated Zeemann splittings in applied magnetic fields shown in Figure 2 (*right*) convey the information on energy gaps with relevance to the magnetic properties. As the thermal occupation factors of these low-energy levels are dependent on the temperature and applied magnetic fields (e.g., 8 cm^–1^ ≈ 11 K), it is expected that the thermodynamic properties are affected at low temperatures. Implications on experimental data will be discussed below.

### ^57^Fe Mössbauer Spectroscopy

**Figure 3:**
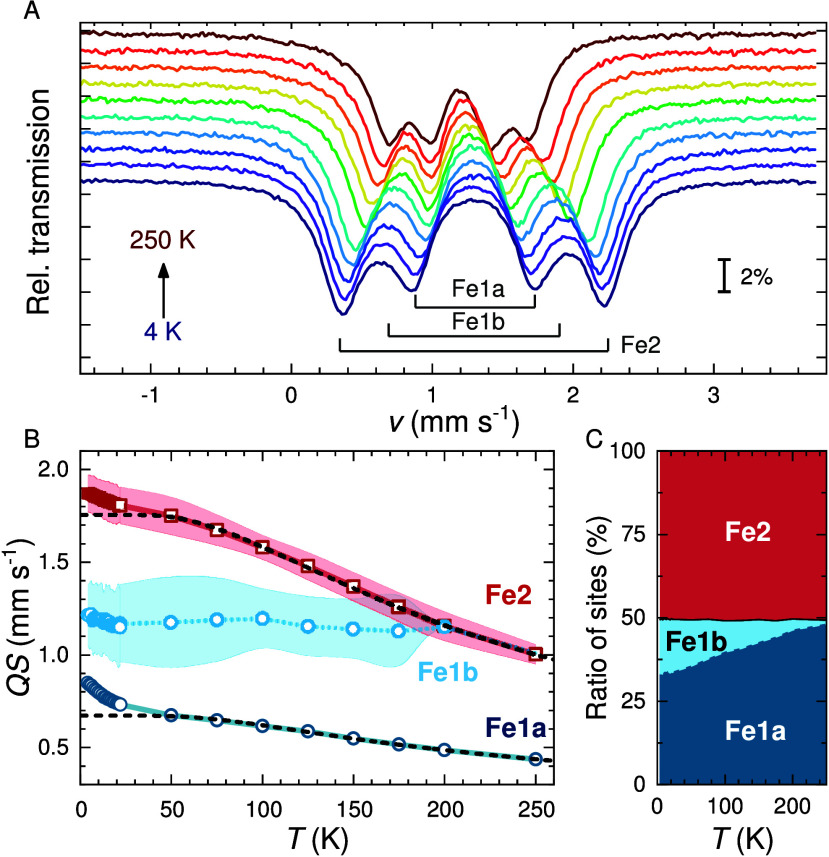
(A) Temperature-dependent ^57^Fe Mössbauer spectra of Cs_2_Fe_2_(MoO_4_)_3_, including references to individual doublet components at 4 K (gray). (B) Quadrupole splitting, *QS*. Shaded areas indicate the width (±σ_*QS*_) of the Gaussian distribution of *QS*. The black dashed curves represent the fits of *QS*(*T*). (C) Spectral contribution of individual Fe-sites.

The temperature-dependent Mössbauer spectra presented in Figure 3 confirm unequivocally two different Fe-sites and the absence of magnetic long-range order (LRO) down to 4 K. These spectra exhibit typical site-specific paramagnetic doublets characteristic of Fe^II^ in the high-spin state (*S* = 2). The main hyperfine parameters derived from fitting procedures of the doublets are (i) the center shift (δ_exp_) related to the oxidation state of iron and the mean square velocity ⟨*v*^2^⟩ of vibrations of the ^57^Fe nucleus (while the intensity of lines relates to the mean square displacement ⟨*x*^2^⟩ due to thermal vibrations) and (ii) the quadrupole splitting (*QS*). The latter arises from the electric field gradient at the ^57^Fe nucleus and is linked to the site-specific ligand field. From the temperature dependence of the center shift (second order Doppler effect) the Mössbauer specific Debye temperatures, θ_M_, are derived as 404(4) K (Fe1) and 308(8) K (Fe2). For further details of hyperfine parameters we refer to the SI and ref (38).

The absence of abrupt changes in the temperature dependence of the hyperfine parameters provides compelling evidence that neither a structural phase transition nor magnetic LRO can be inferred from the presented ^57^Fe Mössbauer spectroscopic data.

At high temperatures two distinct doublets are observed, which indicate the presence of disparate Fe-sites in a 1:1 ratio. We observe *QS* values of 0.44 mm s^–1^ (Fe1) and 1.00 mm s^–1^ (Fe2). These are in line with a degenerate ground state for the former site and a nondegenerate one for the latter. Further support of this assignment stems from the temperature dependence of *QS* above 50 K. We analyzed our data using the three-level model^39^ (eq 1 provided in Materials and Methods), to estimate the approximate electronic excitation energies, Δ_1,2_; see Figure 2. The fitted curves to this model are given as dashed black lines in Figure 3B. For Fe2, we obtain Δ_1_ = 186(4) cm^–1^ and Δ_2_ = 430(10) cm^–1^, consistent with our AOM calculations. The fit of *QS*(*T*) for the dominant Fe1a component provides excitation energies of Δ_1_ = 201(4) cm^–1^ and Δ_2_ = 650(50) cm^–1^. Δ_1_ is approximately independent of the angle Θ (Figure 1B) and in reasonable agreement with the calculated energy difference. This is contrasted by Δ_2_ which is more sensitive to Θ. We infer that with decreasing temperature a further reduction of Θ_avg_ explains the larger Δ_2_-value for Fe1a and refer to additional calculations provided in the SI.

Interestingly, a split component, Fe1b, arises at lower temperatures with spectral contribution provided in Figure 3 C. A larger, nearly temperature-independent *QS* value of ≈1.2 mm s^–1^ is observed for Fe1b, accompanied by a significant Gaussian distribution, σ_*QS*_. The *QS*(Fe1b) value is reminiscent of a nondegenerate ground state; see also Fe2 for comparison. We suggest that the Gaussian distribution, σ_*QS*_ of Fe1b and Fe2, roughly correlates inversely with the energy difference between the nonmagnetic ground state (Γ_1_) and the thermally populated excited magnetic states (Γ_3_); see Figure 2. The feature of Gaussian broadening of spectral lines is absent for Fe1a with a magnetic ground state (Γ_3_).

To summarize the evaluation of our Mössbauer data, we assign the spectral contribution Fe1b to a dynamic lifting of the degenerate ground state of Fe1 subjected to a Jahn–Teller effect on the time scale of the method (≈10^–8^ s). More specifically, the fluctuations associated with these orbital degrees of freedom are governed by the Boltzmann statistics. It follows that with increasing temperature such fluctuations shift away from the Mössbauer time window to shorter characteristic times. Consequently, the Fe1 component with hyperfine parameters given by Fe1a prevails at higher temperatures. Such dynamically fluctuating states are typically associated with distortions. Here, these orbital fluctuations remain local and noncooperative, corresponding to an “orbital frustration” scenario.^40,41^

### Magnetic Properties

With respect to the magnetic properties of Cs_2_Fe_2_(MoO_4_)_3_, fast magnetic fluctuations manifest below 20 K (|θ_CW_| = 22 K). Such enhanced short-range correlations are predominantly linked to the Fe1a site as signalized by an increase of *QS*. For Fe2 with a nondegenerate ground state only moderate changes in *QS*-values are observed at low temperatures. We relate this difference to the reduced (gradually vanishing) magnetic moment for Fe2. As correlated magnetic fluctuations develop into short-range order (see also diffuse scattering observed in neutron experiments provided in the SI), polarization effects—in particular between the two sublattices—might be considered. An estimate of the very weak dipole–dipole interaction (SI) suggests that these do not play a significant role above 4 K.

**Figure 4:**
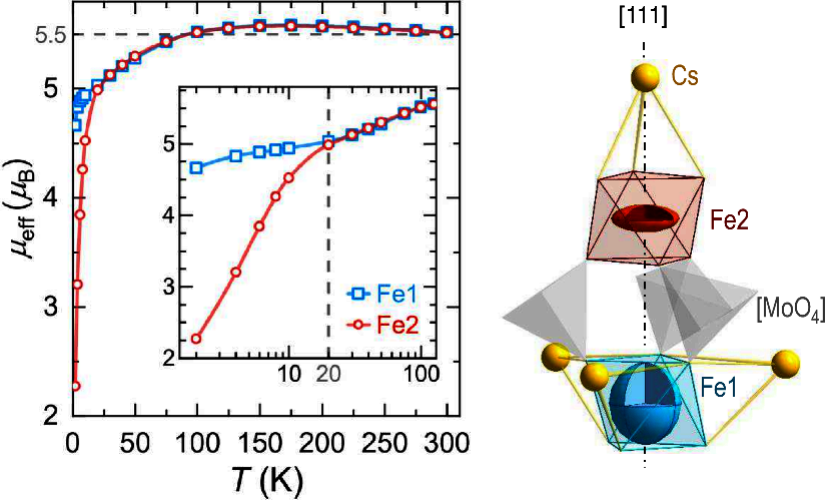
Calculated effective magnetic moments for Cs_2_Fe_2_(MoO_4_)_3_ per Fe-site (AOM). The inset emphasizes the low-temperature region and gray dashed lines serve as a guide to the eye. Single-ion anisotropies (magnetic moments at 4 K) are depicted by ellipsoids with principal axes of 0.1 Å representing 1 μ_B_.

**Figure 5:**
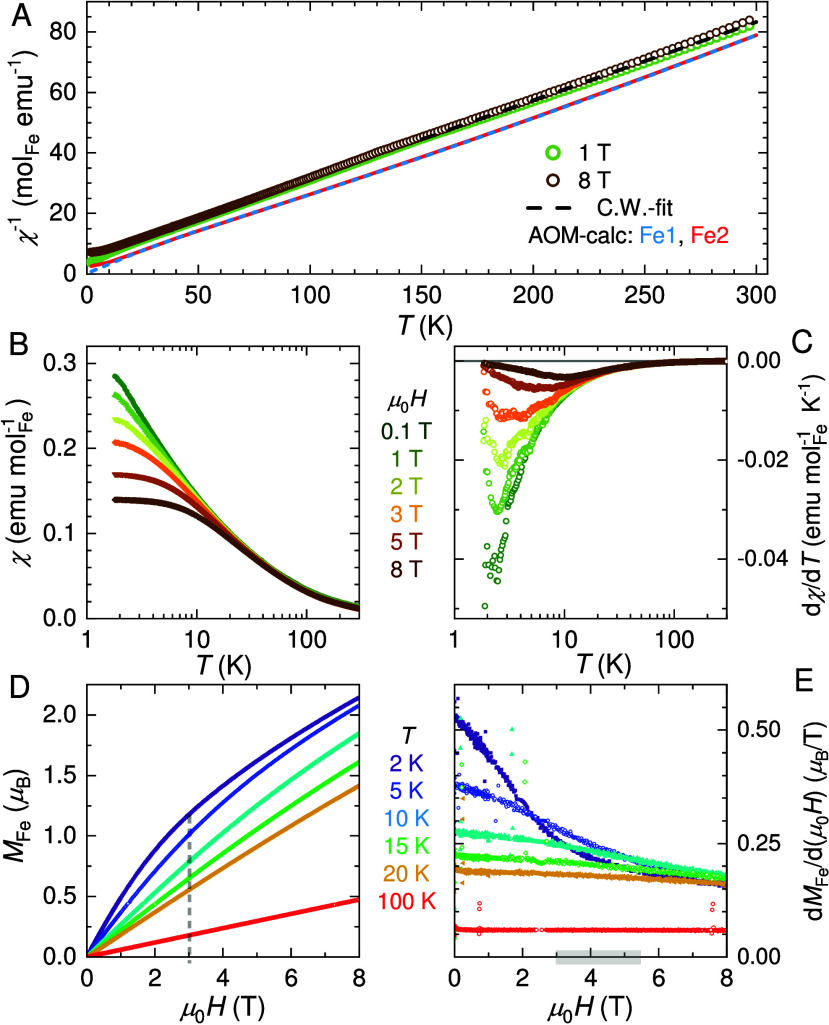
Magnetic data for Cs_2_Fe_2_(MoO_4_)_3_. Temperature-dependent DC susceptibility per mol Fe: (A) Comparison of calculated and experimental χ^–1^(*T*) data. (B, C) χ(*T*) and temperature derivatives in selected applied fields. (D, E) Field-dependent magnetization curves per Fe and their derivatives at selected temperatures.

The paramagnetic properties of Fe1 and Fe2 are calculated based on the individual ligand fields (AOM). The temperature dependence of the Fe-site specific magnetic moments are shown in Figure 4. The magnetic moment at 300 K, μ_calc._ = 5.51 μ_B_ per Fe-site, is fully consistent with the experimental value of ≈5.5 μ_B_ obtained from a Curie–Weiss fit of our reciprocal susceptibility data (Figure 5A). Note that the effective magnetic moment of ≈5.5 μ_B_ per Fe^II^ indicates substantial orbital admixture and exceeds its spin-only value of 4.9 μ_B_.^42,43^ To highlight the importance of the orbital contribution in high-symmetry systems, we compare the cubic Cs_2_Fe_2_(MoO_4_)_3_ with monoclinic α-FeMoO_4_, which has a lower effective magnetic moment of 5.25 μ_B_ at room temperature.^44,45^

At low temperatures the single-ion anisotropy unfolds, revealing the easy-axis anisotropy of Fe1, with its magnetic moment aligned preferably along the 3-fold axis (one of the ⟨111⟩ directions). Easy-plane anisotropy is established for Fe2, which tends to orient its magnetic moment perpendicular to the ⟨111⟩ direction. Both anisotropies are in line with the respective ground states.^46^ Consequently, Fe1 retains its high-spin magnetic moment also at low temperatures (4.66 μ_B_ at 2 K). On the other hand, the energy levels Fe2 are split by ≈16 cm^–1^ (Figure 2 right), which causes a change of the effective moment below 20 K (2.27 μ_B_ at 2 K), see Figure 4. It implies Fe2 is accommodating a fictitious *S*′ = 1 scenario around 4 K.

The field- and temperature-dependent magnetization data of Cs_2_Fe_2_(MoO_4_)_3_ are provided in Figure 5. We start with the discussion of the reciprocal susceptibility per mol_Fe_ (Figure 5A). For comparison, we show the calculated paramagnetic χ^–1^(*T*) data (AOM) for each Fe-site. From fitting the experimental χ^–1^(*T*) to a Curie–Weiss law in applied fields larger than 1 T, we obtain θ_CW_ ≈ −22(2)K and *g*_eff_ = 2.27(2) in the temperature range of 150–300 K. A large dependence of χ(*T*) on the applied field is observed below |θ_CW_|; see also the derivatives of magnetic susceptibility dχ/d*T* (Figure 5B,C). In this temperature range, magnetic fluctuations persist (see also SI for diffuse scattering detected by neutron experiments) which cannot be explained solely by Zeeman splitting for individual Fe^II^. Hence, we ascribe this regime to a cooperative paramagnetic phase with geometric frustration/competing interactions (see Neutron Diffraction studies below) and orbital fluctuations. A further consequence of the disparate single-ion anisotropy is that the perpendicular easy directions of the two Fe-sites (Figure 4) effectively reduce the Heisenberg exchange for *nn* Fe1–Fe2 exchange, *J*_1_. DMI is therefore expected to gain importance in this case. At higher magnetic fields, χ(*T*) indicates that an opening energy gap shapes the magnetic response. For *T* ≤ 5 K the susceptibility approaches a constant value in applied magnetic fields of 8 T (Figure 5B).

The magnetization curves of Cs_2_Fe_2_(MoO_4_)_3_ (Figure 5D) corroborate substantial antiferromagnetic correlations with further information provided in the SI. The derivative, d*M*_Fe_/d(μ_0_*H*), at 2 K approaches a linear field dependence for applied magnetic fields of ∼3.0 T. Above ≈5 T similar slopes for all temperatures below 20 K are observed and reflect the Zeeman splitting for the disparate ground states (Figure 5 E).

### Neutron Diffraction

**Figure 6:**
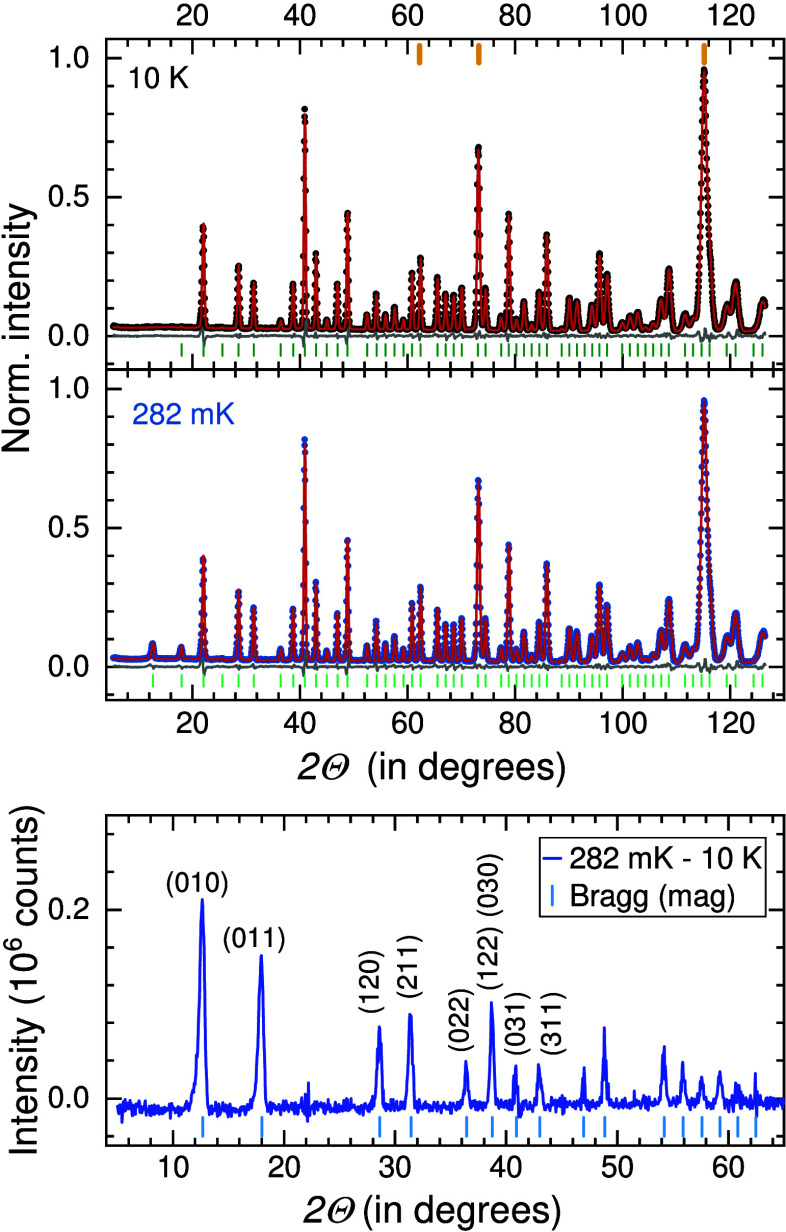
Rietveld refinements of neutron diffraction data (dots) for Cs_2_Fe_2_(MoO_4_)_3_ at 10 K and 282 mK using a wavelength centered at λ = 2.40587 Å. Fits are represented by red lines and differences by gray lines. Markers indicate Bragg positions for the nuclear structure (green) at 10 K, for the refined phase (light green) at 282 mK, and from the Al-container (orange). The bottom panel shows the magnetic contribution to the nuclear data at 282 mK along with marked Bragg positions corresponding to ***k*** = (0,0,0).

LRO is absent down to 2 K in χ(*T*) and *M*(*H*) for Cs_2_Fe_2_(MoO_4_)_3_. In order to gain insights into this frustrated magnet, we performed neutron diffraction studies down to 300 mK; see the SI for further details. Diffuse scattering is observed between 2 and 10 K while LRO develops below *T*_N_ ≊ 1.0 K with an order parameter of β = 0.32(1) indicating 3D-*Ising* classification.^47^ In Figure 6 we show Rietveld refinements of neutron diffraction data at selected temperatures (*R*_nuclear_ = 1.48 and *R*_mag_ = 4.68). The structure solution is obtained in the magnetic space group *P*2_1_3 (No. 198.9) with the magnetic propagation vector, ***k*** = (0,0,0). The bottom panel of Figure 6 provides information on the Bragg positions of the magnetic structure and intensities obtained by subtracting the 10 K data from the 282 mK data. The frustration parameter *f* = |θ_CW_|/*T*_N_ ≊ 22 is large as expected for a noncollinear structure based on equilateral triangles.

**Figure 7:**
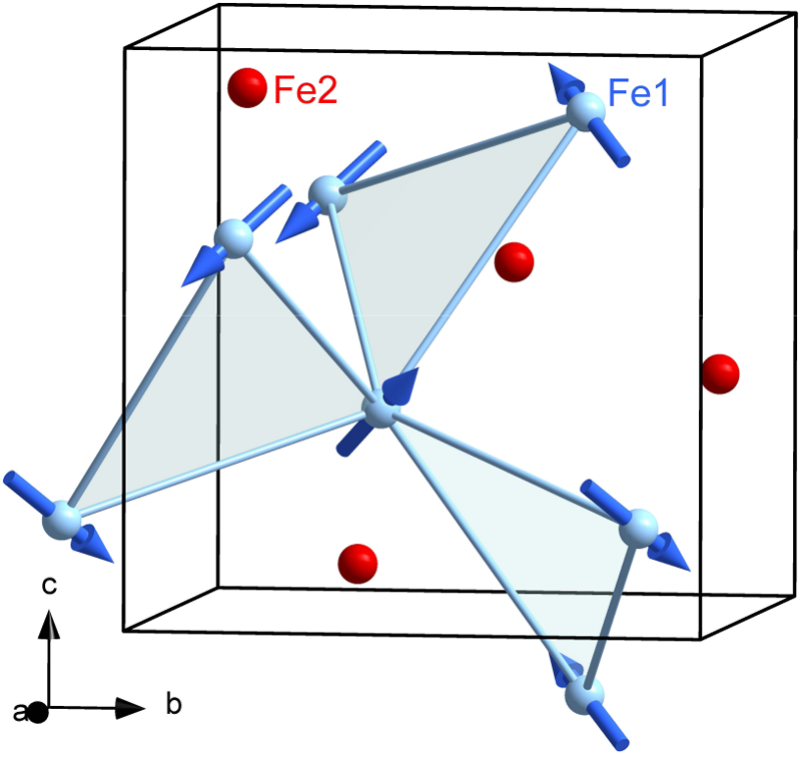
Refined magnetic structure of Cs_2_Fe_2_(MoO_4_)_3_ at 282 mK in the magnetic space group *P*2_1_3 (No. 198.9), propagation vector ***k*** = (0,0,0). The magnetic moments of Fe1 (2.97 μ_B_) and Fe2 (0.17 μ_B_, not shown) align ferromagnetically along ⟨111⟩ for all nearest-neighbors (*nn*).

In Figure 7 we show the magnetic structure of Cs_2_Fe_2_(MoO_4_)_3_ (*P*2_1_3 No. 198.9). The corresponding ordered magnetic moment of Fe1 parallel ⟨111⟩ reaches 2.97 μ_B_ at 300 mK and for Fe2 a significantly lower ordered moment of 0.17 μ_B_ is observed. From paramagnetic AOM-calculations we derive 3.16 μ_B_ (Fe1) and 0.23 μ_B_ (Fe2) for the respective magnetic ground states at 1.0 K in reasonable agreement. We infer that in the LRO phase all (Fe2–Fe1) and (Fe2–Fe2) coupling renders effectively insignificant due to the very low moment of Fe2. Hence, the dominant antiferromagnetic (AFM) exchange originates from a single trillium lattice (Fe1) and relates mainly to *J*_5_ (Fe1). This marks the difference to the isotropic Heisenberg exchange reported for K_2_Ni_2_(SO_4_)_3_ for which only *J*_4_(Ni1–Ni2), corresponding to *J*_3_ for Cs_2_Fe_2_(MoO_4_)_3_, and *J*_5_(Ni1–Ni1) are relevant.^23^ These authors explicitly point out that the *J*_5_-only case corresponds to the single-trillium lattice.

Theoretical work^20^ reports the AFM magnetic ground state of a single trillium lattice based on a classical rotor model with a wave vector (,0,0) featuring 120° rotated spins on each triangle. For Ising systems with the anisotropy axis aligned with the ⟨111⟩ directions, ***k*** = (0,0,0) has been discussed as well.^20^ In reference to a theoretical transition temperature for the single-trillium lattice, *T*_c_ = 0.21(1) |*J*|,^25^ we obtain *J*_5_ = −4.75 K. With respect to frustration phenomena, it has been concluded that the large number of almost degenerate states for order vector orientations obtained from the rotor model should become accessible at temperatures *T* ≥ 0.4 |*J*|,^20^ providing an estimate of *T* ≳ 2.0 K for Cs_2_Fe_2_(MoO_4_)_3_.

### Specific Heat

**Figure 8:**
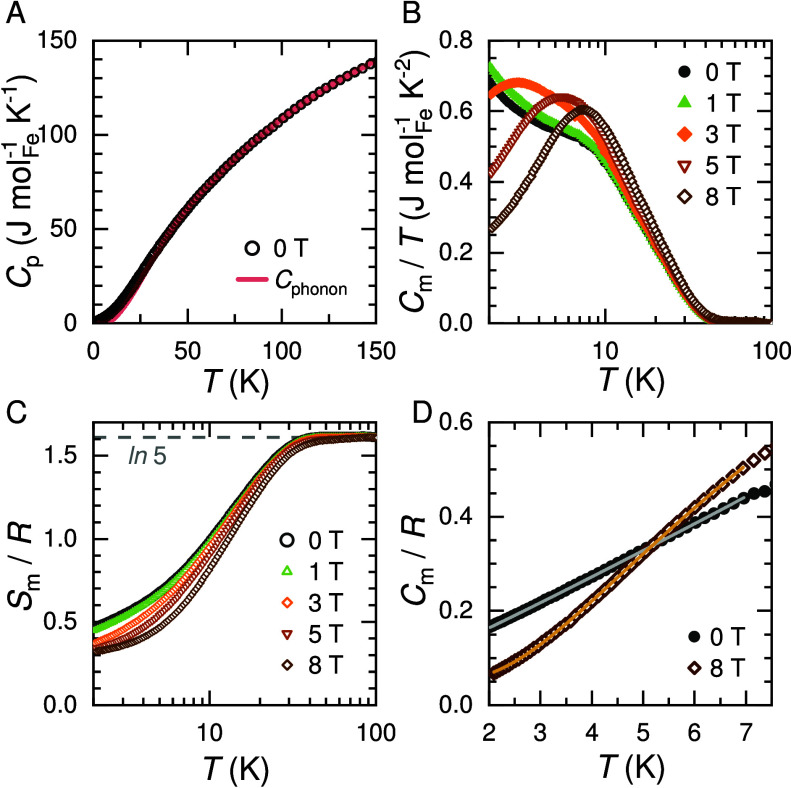
Temperature dependence of the heat-capacity per mol Fe above 2 K in applied magnetic fields for Cs_2_Fe_2_(MoO_4_)_3_. (A) Total zero-field heat capacity and fitted phonon-only contribution, (B) magnetic heat capacity divided by temperature, and (C) magnetic entropy shifted to the theoretical spin-only limit for *S* = 2 at 100 K shown as a dashed line. (D) Low-temperature *C*_m_/*R* data with fits to eq 2 (0 T, gray line) and eq 3 (8 T, orange line). *R* represents the gas constant.

The temperature-dependent heat capacity data of Cs_2_Fe_2_(MoO_4_)_3_ are shown in Figure 8. The magnetic contribution *C*_m_(*T*) to the heat capacity was calculated by subtracting a phonon-only fit based on the semiempirical Debye–Einstein integral.^48^ In order to distinguish between magnetic correlations and a purely paramagnetic scenario, we derived the Schottky anomaly contributions to the heat capacity^49,50^ for the two Fe-sites based on our ligand field calculations (Figure 2). Both procedures are described in the SI in more detail.

A shoulder around 10 K in *C*_m_(*T*)/*T* is observed in zero field (Figure 8B). We attribute this feature to a Schottky anomaly associated with Fe2, where the lowest excitation energy remains relatively constant under applied fields (Figure 2). Upon increasing magnetic fields beyond 3 T, a gap gradually opens with a weight shift of the corresponding contribution to *C*_m_(*T*) from low temperatures toward 10 K. Following the Boltzmann statistics as described by eq 3 we derive an energy gap Δ*E*_g,8T_ ≈ 6.4 cm^–1^ (≈ 9.2 K). This value matches quite closely the calculated maximum in the Schottky anomaly for Fe1 resulting from Zeeman interaction; see also Figure 2 and Figure 5E.

However, magnetic fluctuations and thermal excitations within the energy levels of Fe1 govern the magnetic heat capacity in zero field below |θ_CW_|. *C*_m_(*T*)/*T* is nearly independent of applied magnetic fields of less than 3 T. We observe an almost linear temperature dependence of *C*_m_(*T*)/*R* below 7 K (Figure 8D), as derived from a power-law fit (eq 2 with α ≈ 1.05) in zero field. We suggest the presence of correlated spin fluctuations, soft collective modes,^41^ with rather constant low excitation energy in line with continuous spin excitations (case II) discussed for K_2_Ni_2_(SO_4_)_3_.^23^ Overall, for the strongly frustrated magnet Cs_2_Fe_2_(MoO_4_)_3_ short-range magnetic correlations dominate below |θ_CW_| and above *T*_N_.

The magnetic entropy *S*_m_(*T*) of Cs_2_Fe_2_(MoO_4_)_3_ (Figure 8C) is obtained from integration of *C*_m_(*T*)/*T* between 2 and 150 K. Since none of the *C*_m_(*T*)/*T* curves shown in Figure 8B reaches zero at 2 K, the derived magnetic entropy is incomplete even for the highest applied field. However, the *S*_m_(*T*) data settle into a plateau, similar to the nearly isotropic magnetic moments at high temperatures (Figure 4). Accordingly, we shifted our experimental *S*_m_(*T*) data to the corresponding theoretical value *R* ln(2*S* + 1) with *S* = 2 at 100 K. The remaining entropy in the system below 2 K is then of the order of 30% in zero field corresponding to (i) gradual changes from *S* = 2 to *S*′ = 1 for Fe1 and from *S*′ = 1 to *S*′ = 0 for Fe2 according to the respective ground states (Boltzmann statistics) and (ii) the energy contribution related to the λ-anomaly upon LRO.

The in depth characterization of Cs_2_Fe_2_(MoO_4_)_3_ reveals interesting properties in a temperature regime below |θ_CW_| = 22 K, such as enhanced degeneracy of the magnetic ground state perturbed by the orbital degrees of freedom and a significant magnetic entropy release in response to applied magnetic fields. Thus, our motivation extends to the investigation of Cs_2_Fe_2_(MoO_4_)_3_ as a potential magnetic coolant in a temperature regime of up to *T* ≈ 20 × *T*_N_ and therefore of interest for hydrogen liquefaction.

### Magnetocaloric Effect

**Figure 9:**
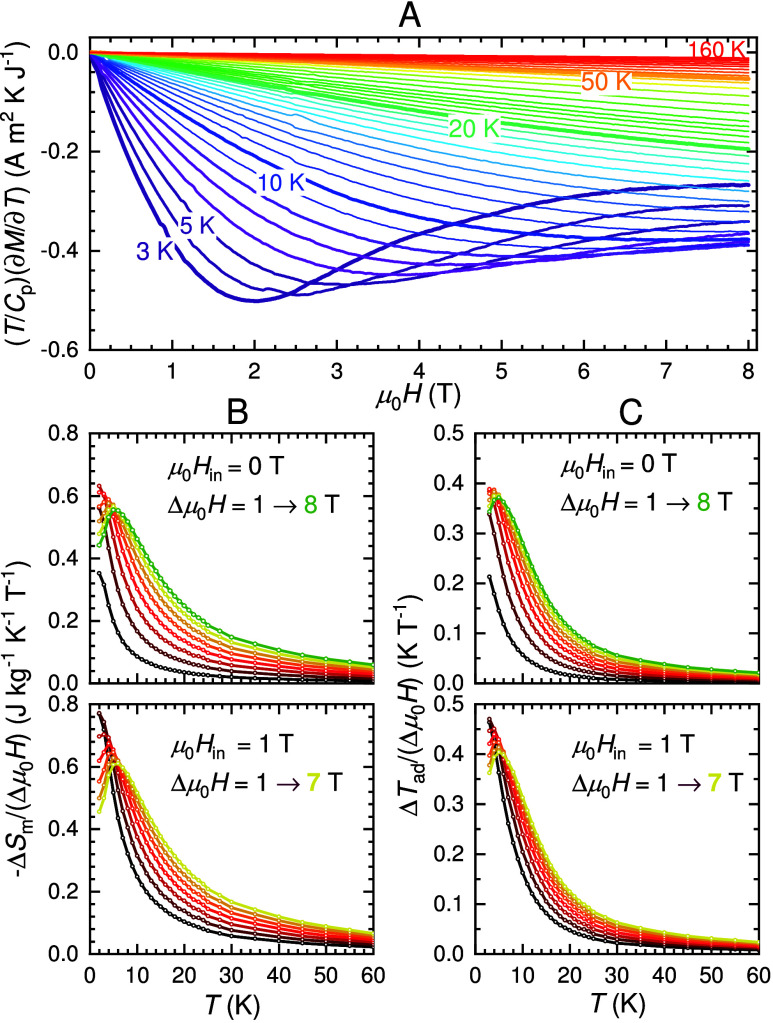
Magnetocaloric parameters for Cs_2_Fe_2_(MoO_4_)_3_. (A) Isotherms for the temperature derivative of the magnetization with respect to the total specific heat data. Normalized isothermal magnetic entropy change (B) and adiabatic temperature change (C) given for initial magnetic fields of μ_0_*H*_*in*_ = 0 T (top) and 1 T (bottom). Data are shown for increments of Δ*μ*_0_*H* of 1 T.

The magnetocaloric (MC) parameters, i.e., the total isothermal magnetic entropy change −Δ*S*_m_, the adiabatic total temperature change Δ*T*_ad_, and relative cooling power *RCP*, were calculated from eq 4 and eq 5, respectively, based on indirect measurements as described in Materials and Methods. In Figure 9A we show the field dependence of the integrand for Δ*T*_ad_ (eq 5) visualizing the combined magnetization and specific-heat data for various temperatures. Figure 9B,C illustrates −Δ*S*_m_ and Δ*T*_ad_ normalized to the magnetic field change, Δ*μ*_0_*H*.

In the temperature range below ≈10 K, we recall the presence of two regions: (i) a “gapless” ground state below μ_0_*H* ≈ 3 T and (ii) a “gapped” ground state above μ_0_*H* ≈ 3 T. These give rise to a maximum in the MC parameters. According to the power law, –Δ*S*_m_ ∝ *H*_fin_^*n*^, we derived from −Δ*S*_m_(*T*, *H*) curves in the respective regions (i) *n*_*T*_1__ ≈ 1.53 and *n*_*T*_2__ ≈ 1.93 and (ii) *n*_*T*_1__ ≈ 0.05 and *n*_*T*_2__ ≈ 1.25 for *T*_1_ = 2 K and *T*_2_ = 10 K; see the SI. The paramagnetic state above 100 K is characterized by *n* = 2. Such a large difference in *n*(*T*) underlines the distinct origin of the MC effect in the two regimes.

Intriguing are the MC parameters within the gapless phase (i). As can be inferred from Figure 9, we observe no maximum in this case, but a large dependence on the initial applied magnetic field. The low MC values obtained for μ_0_*H*_in_ = 0 T upon field change of 1 T indicate a minor sensitivity of the low-energy spin excitations to small applied magnetic fields. In comparison, Δ*T*_ad_ and −Δ*S*_m_ increase by a factor of ≈2.2 for an initial field μ_0_*H*_in_ = 1 T upon a field change of 1 T at 2 K. Within (i), the normalized MC response is efficient over a narrower temperature range but is relatively high under a small change in applied fields. This enhanced performance relates to a combined effect due to Zeeman splitting and the concomitant suppression of continuous spin excitations upon a transition from (i) to (ii) linked to Fe1.

As the final applied field increases within the gapped (ii) region, continuous spin excitations are increasingly suppressed and the normalized MC parameters exhibit only a weak dependence on the initial field. Concurrently, the enhanced MC response of Cs_2_Fe_2_(MoO_4_)_3_ expands to higher temperatures. The maxima of the MC parameters shift with final applied fields to higher temperatures up to *T*_max_ ≈ 5 K upon Δ*μ*_0_*H* = 8 T. Importantly the MC parameters remain significant beyond 20 K and reach −Δ*S*_m,max_ = 4.4 J kg^–1^ K^–1^ and Δ*T*_ad,max_ = 3.0 K. The *RCP* attains 79 J kg^–1^ for Δ*μ*_0_*H* = 8 T.

These parameters are moderately lower compared to selected rare-earth containing molybdates below their spin-glass transition temperatures, e.g., *RE*_2_Mo_2_O_7_ with *RE* = Er, Dy, and Gd, with −*ΔS*_m,max_(7 T, ≈ 10 K) = 12–15 J kg^–1^ K^–1^.^51^ The double perovskite Tb_2_FeCrO_6_ orders antiferromagnetically at 8.5 K and exhibits a MC effect with −Δ*S*_m,max_(0 to 7 T) = 12.9 J kg^–1^ K^–1^.^52^ In contrast to such traditional rare-earth containing magnetocaloric materials based on cooling in the vicinity of a rather sharp phase transition, strongly frustrated magnets ensure enhanced magnetocaloric parameters smoothly changing over a broad temperature range. For the trillium lattice, Na[Mn(HCOO)_3_], MC parameters have been derived for a local maximum in the vicinity of a magnetic disorder–order transition at 220–290 mK, above *T*_N_.^21^ We emphasize that in our case a global maximum of −Δ*S*_m_ is reached at an order of magnitude higher temperature.

## Concluding Remarks

To conclude, we have reported the properties of Cs_2_Fe_2_(MoO_4_)_3_ with respect to single-ion anisotropies of the two crystallographically independent Fe-sites forming a highly frustrated lattice of the interlinked double-trillium lattice. We found two disparate ground states from ligand-field calculations and ^57^Fe Mössbauer spectroscopic data. The predominantly antiferromagnetic correlations (θ_CW_ ≈ −22 K) are dressed with orbital frustration arising from the inherent easy-axis and easy-plane anisotropies of Fe^II^. Magnetic LRO occurs at *T*_N_ ≊ 1.0 K. The magnetic structure with ***k*** = (0,0,0) obtained from neutron diffraction data corresponds to the dominant exchange related to the Fe-site with easy-axis anisotropy and a degenerate ground state. We point out that this is an experimental realization of a theoretical model for the trillium lattice and documents the difference in propagation vectors for Heisenberg and Ising systems. From the analysis of the magnetic heat capacity we find that the system features low-energy—possibly gapless—magnetic excitations in zero or low applied magnetic fields, whereas for μ_0_*H* ≥ 3 T a gap opens at low temperatures related to Zeeman interaction.

The magnetocaloric response is associated with the disparate ground states of Fe ions in each trillium sublattice. The material should be suitable to serve as a precooling stage across a broad temperature range around 20 K and below, where correlated magnetic fluctuations dominate. The latter drives the cooling performance to even lower temperatures in this frustrated magnet.

To summarize, compounds belonging to the frustrated trillium lattice exhibit attractive magnetocaloric parameters and offer intriguing prospects for cryogenic cooling, especially upon small changes in magnetic fields. The inherent properties of such highly correlated systems with low excitation energies between their nearly degenerate ground states ensure the dissipation of latent heat associated with liquefaction, thereby minimizing material degradation. Moreover, this rare-earth-free cubic material, whose performance in magnetic refrigeration does not rely on magneto-structural phase transitions, is likely to provide lower mechanical wear and cohesiveness over refrigeration cycling. This material serves as a proof of concept for using highly frustrated magnets, hosting distinct magnetic sites with nearly degenerate ground states, over a wider temperature range.

## Materials and Methods

### Synthesis

The precursor Cs_2_MoO_4_ was obtained from reacting Cs_2_CO_3_ (99%, Alfa Aesar) and MoO_3_ (99.9+%, ChemPur, fine chemicals) at 600 °C for 48 h in a corundum crucible. FeMoO_4_ (mainly α-phase) was prepared according to ref (45) by a metallothermic reduction of Fe_2_O_3_ (99.9%, ChemPur, fine chemicals) with iron powder (99.9%, ChemPur, fine chemicals) in the presence of MoO_3_ at 800 °C for 48 h in evacuated (*p* ≈ 2–4 × 10^–2^ mbar) sealed silica glass ampules. Microcrystalline Cs_2_Fe_2_(MoO_4_)_3_ was prepared by a solid state reaction of Cs_2_MoO_4_ and FeMoO_4_ in the molar ratio of 1:2 at 600 °C for 7 d in evacuated (*p* ≈ 2–4 × 10^–2^ mbar) sealed silica glass ampules.

### X-ray Diffraction

Powder X-ray diffraction data were collected at room temperature in transmission geometry on a STOE Stadi P diffractometer (STOE & Cie) using Mo Kα_1_ radiation (λ = 0.7093 Å) and a Dectris MYTHEN 1K detector. The sample was placed between two polyvinyl acetate foils on a flat sample holder, and NIST SRM 660c (LaB_6_) was used as an external standard. The lattice constants of Cs_2_Fe_2_(MoO_4_)_3_ were determined by capillary (diameter 0.3 mm, thickness 0.01 mm) measurements. The Rietveld analysis of the diffraction data was performed using the TOPAS-Academic software, version 7.20.^53^ For the visualization of the structure, the Diamond software (Crystal Impact, ver. 4.3.2) was employed. To analyze the coordination sphere of Fe, a dummy atom (D) was placed in the center of each equilateral triangular face formed by the crystallographically independent O atoms. These dummies were used to determine the Θ = ∠(D–Fe–O) angles.

### Angular Overlap Model Calculations

The angular overlap model (AOM)^37,54^ presents a rational choice to evaluate the electronic properties of 3d-metal complexes including spin–orbit coupling. The model Hamiltonian of AOM provides a fast and qualitatively correct interpretation for a mere fraction of computational resources compared to *ab initio* methods, which require a complex representation of the electronic ground and excited-state wave functions.^55^ AOM calculations were performed using the CAMMAG suite.^35,36,56^ CAMMAG allows calculations of transition energies, magnetic moments and susceptibility, etc., from bonding parameters within a complex for which the metal and ligand coordinates are obtained from the structure refinement.

For our calculations, we used the Racah parameters *B* = 718 cm^–1^ and *C* = 3085 cm^–1^, the spin–orbit coupling parameter |λ| = ζ/2*S* with ζ = 400 cm^–1^, and isotropic bonding parameters *e*_σ_ = 4250 cm^–1^ and *e*_π_ = 1000 cm^–1^, which relate to the octahedral ligand-field splitting parameter Δ_o_ = 3*e*_σ_ – 4*e*_π_.^37^ The isotropic orbital reduction factor accounting for the reduction of orbital momentum contribution to the magnetic moment due to electron delocalization (covalence) was set to a typical value for 3d-metal oxides, *k* = 0.8.^37,57^ The limit taken for thermal occupancy was 1400 cm^–1^ and for second order Zeeman interactions 12000 cm^–1^.

### ^57^Fe Mössbauer Spectroscopy

Transmission Mössbauer spectra of microcrystalline Cs_2_Fe_2_(MoO_4_)_3_ were collected using a ^57^Co(Rh) source in a custom-built setup including a closed-cycle cryostat C2 by Montana Instruments, in the temperature range of 4–250 K in zero field. Calibration of the isomer shift and velocities was performed with respect to a standard α-Fe foil. The absence of a preferred orientation was confirmed at selected temperatures by comparing the spectra to those collected at the magic angle, i.e., when the normal vector of the sample plane and the wave vector of γ-rays form an angle of 54.7°.^58^

The Mössbauer spectra of Cs_2_Fe_2_(MoO_4_)_3_ were analyzed with the Recoil software^59^ by using the Extended Voigt-based Fitting (xVBF) analysis^60^ to extract the Gaussian distribution of the quadrupole splittings and hyperfine magnetic fields for each Fe-site. The full width at half-maximum of the Lorentzian natural line was fixed to 0.28 mm s^–1^. The spectra contain also a doublet with very low intensity originating in the experimental setup (Fe admixtures in the detector’s Be window, kept at room temperature), whose hyperfine parameters are constant over the whole measured temperature range and thus fixed during fitting (δ_exp_ = 0.198 mm s^–1^, *QS* = 0.295 mm s^–1^).

The temperature dependence of *QS* in compounds with high-spin Fe^II^ in distorted octahedral coordination is dictated by the temperature-dependent occupation of the excited states. Therefore, *QS*(*T*) is analyzed by using a three-energy-level model (eq 1) introduced by R. Ingalls,^39^ with energies of Δ_1_ and Δ_2_ above the ground state:
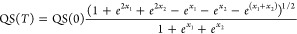
1with
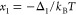
and
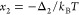
Here *QS*(0) refers to the extrapolated value for *T* → 0 K and *k*_B_ to the Boltzmann constant. For further analysis of the chemical shifts and hyperfine magnetic fields, we refer to the SI materials.

### Neutron Diffraction

Neutron diffraction was measured on a HB-2A powder diffractometer at the High Flux Isotope Reactor (HFIR) at Oak Ridge National Laboratory. The well-ground sample (≈8.2 g) was mounted into an Al container with the diameter of 0.25 in. in a He-glovebox and placed in a ^3^He cryostat. The data were collected in the temperature range of 0.3–50 K with the incident neutron wavelength centered around λ = 2.40587 Å. The counting times were ≈6 h for 282 mK, 700 mK, 2 K, 10 K, and 50 K and 22 min for the fast scans in 0.1 K increments between 0.3 and 1.4 K. The data were analyzed in the FullProf suite, and the propagation vector of the magnetic structure was determined by using the functionality ***k***-Search.^61^

### Magnetic Measurements

Magnetic data were measured by using the vibrating sample magnetometry (VSM) option of the cryogen-free Physical Property Measurement System (PPMS DynaCool 9) by Quantum Design (QD). A piece of a pressed pellet of Cs_2_Fe_2_(MoO_4_)_3_ was wrapped in a polyethylene foil and placed in a polypropylene sample holder (QD). The direct current (DC) susceptibility, approximated by χ = *M*/*H*, was measured from 2 to 300 K in selected applied magnetic fields between 10 mT and 8 T, with the logarithmic spacing between the points to emphasize the low-temperature region. The molar susceptibility per Fe was corrected for diamagnetic contributions (−1.26 × 10^–4^ emu mol^–1^). The temperature derivative of the DC susceptibility presented in Figure 5B was smoothed by an 11-point adjacent averaging procedure. Isothermal field-dependent DC magnetization curves from 0 T up to 8 T were measured with temperature increments of 1 K (from 2 to 25 K), 2.5 K (to 30 K), 5 K (to 60 K), 10 K (to 100 K), 20 K (to 200 K), and 25 K (to 300 K), for the calculation of MC parameters. Missing data from the touchdown procedure, used for the sample centering, produced a kink in the *M*(*H*) data at around 2–2.4 T, and were replaced using the Akima spline interpolation in Figures 5C,D and 9A.

### Heat Capacity

The heat capacity data of Cs_2_Fe_2_(MoO_4_)_3_ were obtained by using the PPMS heat capacity option (QD). Prior to the actual measurements, an addenda was measured for the grease and puck platform correction. The isobaric heat capacity measurements were performed by the standard relaxation method in the temperature range of 2–150 K and in applied magnetic fields from 0 up to 8 T (*p* = 10^–5^ mbar). The heat capacity was measured three times at each temperature point, while logarithmic spacing between the temperature points was employed. In order to estimate the phonon contribution to the experimental heat capacity at zero field, we used the Debye–Einstein integral modified from ref (48). For further details on the fitting procedure see the SI. The phonon contribution *C*_phonon_ was then subtracted from the experimental heat capacity data *C*_p_ to obtain the magnetic part *C*_m_. Magnetic entropy was calculated by integrating *C*_m_/*T* from 2 to 150 K.

The low-temperature magnetic heat capacity was fitted in the temperature range of 2–7 K by a power law (eq 2)
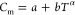
2and a model (eq 3) simulating an energy gap *ΔE*_g_
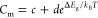
3Here, *a*, *b*, α, *c*, *d*, and Δ*E*_g_ are the fitting parameters; see the SI.

### Magnetocaloric Parameters

The total isothermal magnetic entropy change (eq 4) was calculated based on the isothermal *M*(*H*) curves
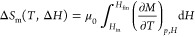
4The adiabatic total temperature change (eq 5) was derived from
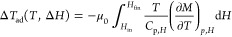
5which combines both the magnetic and heat capacity data to avoid approximating the zero-temperature entropy.^62,63^ In these equations, *H*_in_ and *H*_fin_ denote the initial and final magnetic field with Δ*H* = *H*_fin_ – *H*_in_.

For the integration, numerical derivatives ∂*M*(*T*, *H*)/∂*T* based on the *M*(*H*) curves and 0.05 T increments were used. In comparison to the magnetization, the heat capacity varies rather marginally with the field, therefore the field dependence of *C*_p_/*T* was approximated by Akima-spline interpolation based on the measured *C*_p_(*T*) curves at various fields.

The relative cooling power is usually defined as *RCP* = −Δ*S*_m,max_·*ΔT*_fwhm_, where Δ*T*_fwhm_ is the full width of −Δ*S*_M_ at half-maximum. Because our data do not cover the whole range of the −Δ*S*_M_ peak, we approximated Δ*T*_fwhm_ by the temperature at which −Δ*S*_M_ reaches the half-maximum value.

## Supporting Information Available

Additional experimental details, materials, methods, and figures for X-ray diffraction data, neutron diffraction data, AOM calculations, dipole–dipole interaction, ^57^Fe Mössbauer spectroscopy, heat capacity data, magnetization data, and magnetocaloric effect (PDF)
