# Supplementary material for: Cs2Fe2(MoO4)3—A Strongly Frustrated Magnet with Orbital Degrees of Freedom and Magnetocaloric Properties
Source: Chem Mater. 2024 Jul 2;36(14):7016–25. doi: 10.1021/acs.chemmater.4c01262 (PMC11270738; doi:10.1021/acs.chemmater.4c01262)
Supplement: Supplementary file 1 — cm4c01262_si_001.pdf [file cm4c01262_si_001.pdf]

$Cs_2Fe_2(MoO_4)_3$ – a Strongly Frustrated Magnet with Orbital Degrees of Freedom and Magnetocaloric Properties

Lenka Kubíčková,[1] Anna Katharina Weber,[1] Martin Panthöfer,[1] Stuart Calder,[2] and Angela Möller[1]

[1] *Department of Chemistry, Johannes Gutenberg University Mainz, Duesbergweg 10-14, 55128 Mainz, Germany*

[2] *Neutron Scattering Devision, Oak Ridge National Laboratory, Oak Ridge, Tennessee 37831, United States*

CONTENTS

X-ray diffraction data 2

Neutron diffraction data 3

AOM calculations 6

Dipole-dipole interaction 8

^{57}Fe Mössbauer spectroscopy 9

Heat capacity data 12

Magnetization data 15

Magnetocaloric effect 16

References 17

Email: angela.moeller@uni-mainz.de

X-RAY DIFFRACTION DATA

A room-temperature powder X-ray diffractogram of the analyzed microcrystalline sample of $Cs_2Fe_2(MoO_4)_3$ is shown in Fig. S1 and crystallographic information obtained from the Rietveld refinement using the Topas suite [1] is summarized in Tab. S1.

TABLE S1. Crystallographic data for $Cs_2Fe_2(MoO_4)_3$ obtained from the Rietveld refinement at room temperature. (Space group $P2_13$, $a = 10.9112(1)$ Å, $Z = 4$, $R_{\mathrm{wp}} = 7.65\,\%$, Bragg $R = 1.60\,\%$.)

Atom	Wyck.	Site	x/a	y/b	z/c	B_{iso}
Cs1	4a	.3.	0.1788(1)	0.3212(1)	0.6788(1)	1.83(5)
Cs2	4a	.3.	0.4578(2)	0.9578(2)	0.5422(2)	1.83(5)
Mo1	12b	1	0.3009(2)	0.6240(2)	0.5262(2)	0.58(5)
Fe1	4a	.3.	0.3373(3)	0.3373(3)	0.3373(3)	0.43(8)
Fe2	4a	.3.	0.6125(3)	0.6125(3)	0.6125(3)	0.43(8)
O1	12b	1	0.276(1)	0.484(1)	0.448(1)	1.4(2)
O2	12b	1	0.455(1)	0.672(1)	0.523(1)	1.4(2)
O3	12b	1	0.251(1)	0.605(1)	0.678(1)	1.4(2)
O4	12b	1	0.207(1)	0.731(1)	0.450(1)	1.4(2)

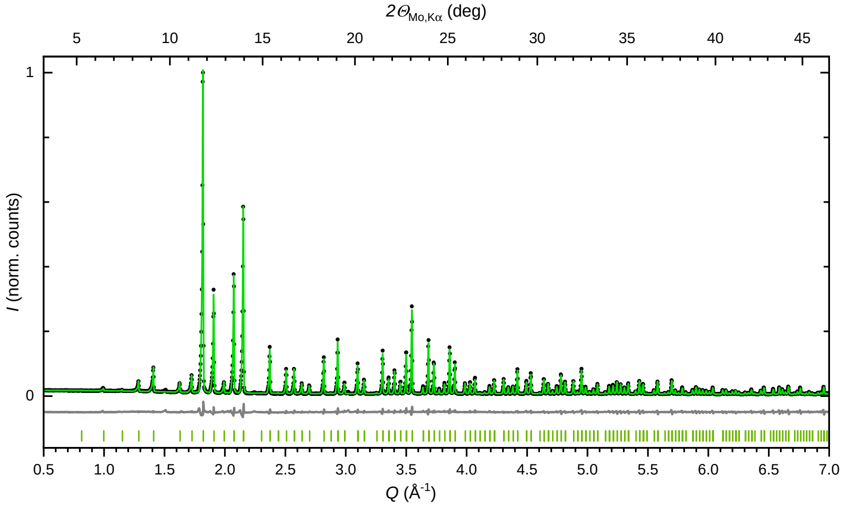

FIG. S1. Rietveld refinement of powder X-ray diffraction data for $Cs_2Fe_2(MoO_4)_3$ at room temperature. Bragg positions are represented by bars, the difference between data (black dots) and the fit (green line) is given by a gray line.

NEUTRON DIFFRACTION DATA

Neutron diffraction data were collected at the HB-2A beamline at ORNL (proposal number IPTS-31426.1) and refined using the Fullprof-suite [2]. Crystallographic data for 10 K are provided in Tab. S2. Pronounced diffuse scattering, indicative of short-range ordering, is observed below 10 K and persists with minimal change down to 2 K, see Fig. S2 for difference in intensities Δ(Int.) at selected temperatures. In Fig. S3 we show the Rietveld refinement of data below T_N. Details of the refinement below 1.1 K are given along with the magnetic moments of individual Fe-sites in Tab. S3. Information on the magnetic space group is provided in Tab. S4, supported by the magnetic CIF format (magCIF). The development of LRO below 1.0 K based on normalized intensities is shown in Fig. S4.

TABLE S2. Crystallographic data for $Cs_2Fe_2(MoO_4)_3$ obtained from the Rietveld refinement at 10 K of neutron diffraction data, $\lambda = 2.406$Å. (Space group $P2_13$, $a = 10.88360(6)$ Å, $Z = 4$, $R_{wp} = 5.31,\%$, $R_{Bragg} = 1.49\,\%$.)

Atom	Wyck.	Site	x/a	y/b	z/c	B_{iso}
Cs1	4a	.3.	0.8211(5)	0.8211(5)	0.8211(5)	0.5(2)
Cs2	4a	.3.	0.0422(4)	0.0422(4)	0.0422(4)	0.6(2)
Mo1	12b	1	0.2998(3)	0.6225(4)	0.5271(4)	0.1(1)
Fe1	4a	.3.	0.3372(3)	0.3372(3)	0.3372(3)	0.3(1)
Fe2	4a	.3.	0.6120(3)	0.6120(3)	0.6120(3)	0.3(1)
O1	12b	1	0.2749(4)	0.4818(5)	0.4511(4)	0.3(2)
O2	12b	1	0.4554(5)	0.6712(4)	0.5160(4)	0.3(1)
O3	12b	1	0.2557(3)	0.6031(4)	0.6810(4)	0.3(1)
O4	12b	1	0.2081(4)	0.7355(4)	0.4516(4)	0.4(1)

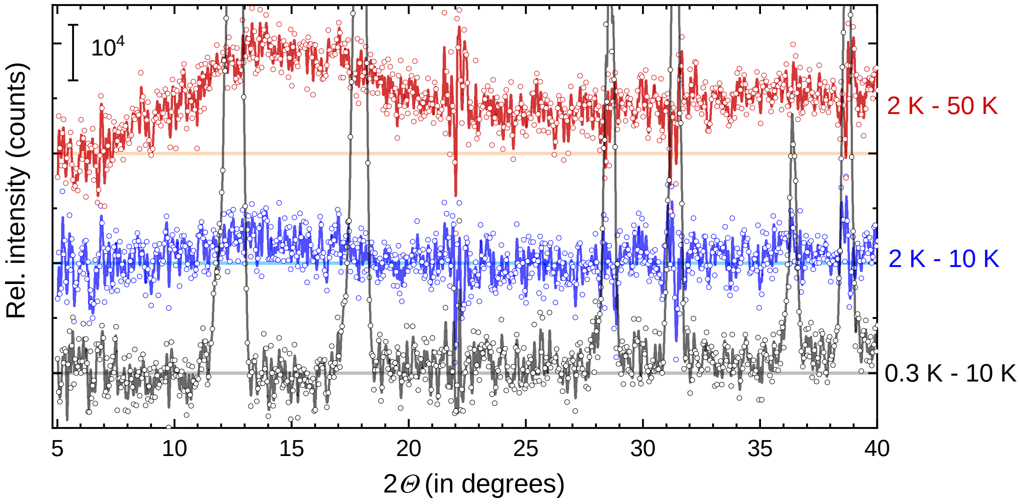

FIG. S2. Diffuse scattering of powder neutron diffraction data (6 h scans, $\lambda = 2.406$Å, HB-2A) for $Cs_2Fe_2(MoO_4)_3$. ΔInt. data are obtained by subtracting the respective diffraction patterns for selected temperatures. Horizontal lines refer to the baselines of the shifted data, respectively.

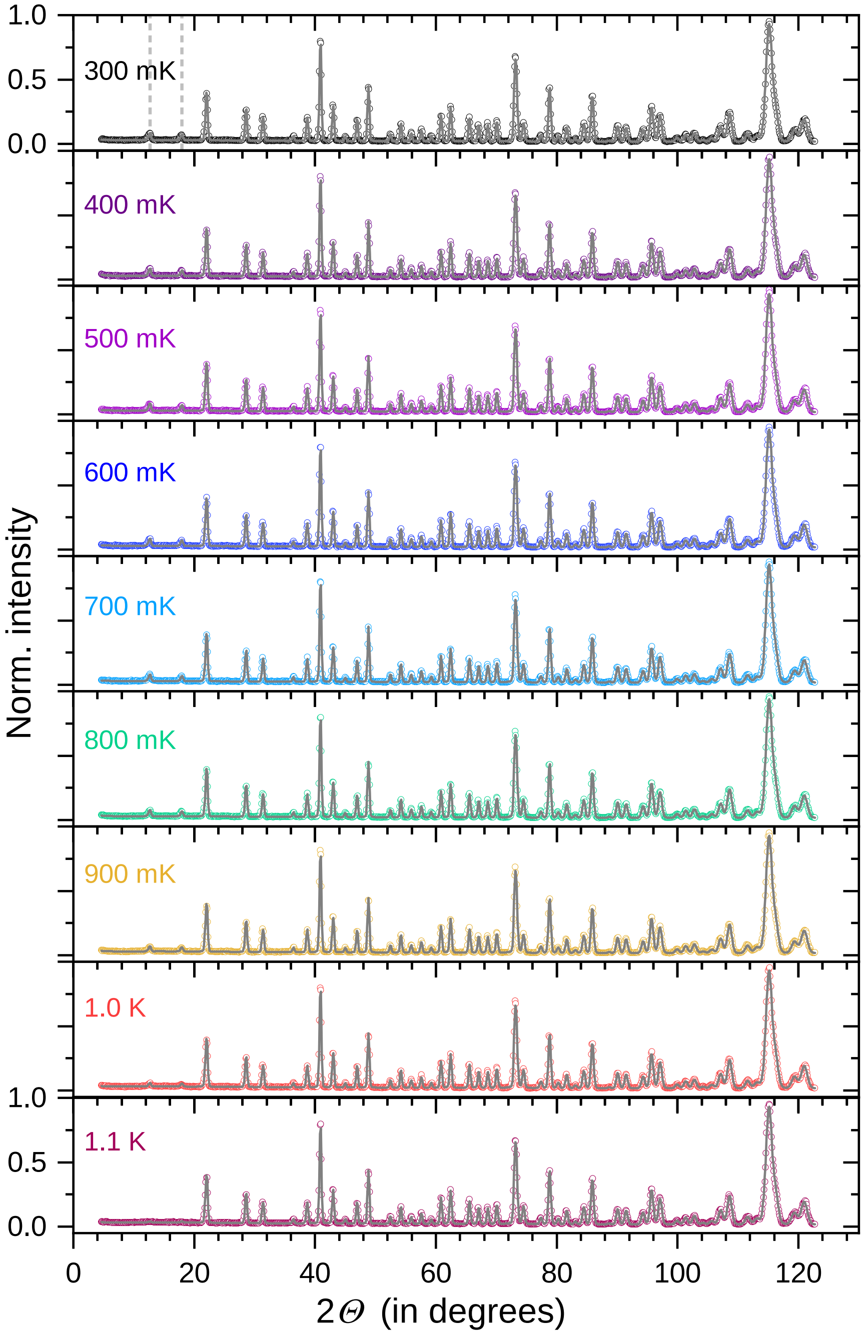

FIG. S3. Refinement of powder neutron diffraction data ($\lambda = 2.406$Å, HB-2A) for $Cs_2Fe_2(MoO_4)_3$ at selected temperatures. Data are shown as dots and the fit by gray lines. The dashed lines refer to two magnetic reflections.

TABLE S3. Lattice constants and magnetic moments for $Cs_2Fe_2(MoO_4)_3$ obtained from the Rietveld refinement of neutron diffraction data (temperature stabilized 22 min scans, $\lambda = 2.406$Å), space group $P2_13$, $R_{\mathrm{Bragg}} = 2.5\%$ and $\chi^2 \approxeq 6.5\%$. R-factors are given for the magnetic phase (R_m) and the overall refinement (R_{wp}).

T (K)	1.1	1.0	0.9	0.8	0.7	0.6	0.5	0.4	0.3
a (Å)	10.8850(2)	10.8848(2)	10.8848(2)	10.8848(3)	10.8847(3)	10.8849(2)	10.8848(2)	10.8846(3)	10.8847(2)
R_m (%)	–	11.5	10.2	6.54	8.43	4.98	6.34	7.59	6.41
R_{wp} (%)	8.90	9.22	9.22	8.94	9.09	9.05	9.05	9.10	9.19
Fe1 (μ_B)	–	2.09(6)	2.50(5)	2.76(5)	2.83(5)	2.95(5)	2.96(5)	2.99(5)	2.97(5)
Fe2 (μ_B)	–	0.22(16)	0.29(15)	0.19(14)	0.18(14)	0.21(13)	0.24(13)	0.16(13)	0.22(13)

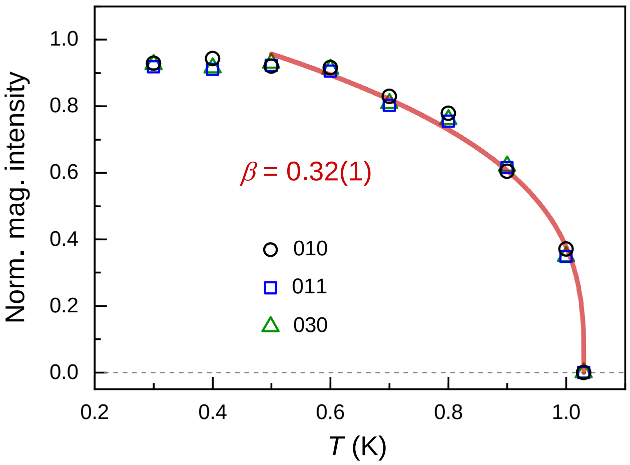

FIG. S4. Temperature dependence of normalized intensities of selected magnetic reflections (λ = 2.406 Å, HB-2A). A power law fit, $I(T) \propto \mid T - T_c \mid^{\beta}$, is given as a red line.

TABLE S4. Magnetic space group (MSG) for $Cs_2Fe_2(MoO_4)_3$ with basic information about its relation with its parent paramagnetic structure.

Parent space group	$P2_13$ (No. 198)	
Propagation vector(s)	(0,0,0)	
Transformation from parent basis to the one used	(**a**,**b**,**c**; 0,0,0)	
MSG symbol	$P2_13$	
MSG number	198.9	
Magnetic point group	23.1 (No. 28.1.107)	
MSG symmetry operations	x,y,z,+1	$\{1\|0\}$
	x+$\frac{1}{2}$,−y+$\frac{1}{2}$,−z,+1	$\{2_{100}\|\frac{1}{2}\frac{1}{2}0\}$
	−x,y+$\frac{1}{2}$,−z+$\frac{1}{2}$,+1	$\{2_{010}\|0\frac{1}{2}\frac{1}{2}\}$
	−x+$\frac{1}{2}$,−y,z+$\frac{1}{2}$,+1	$\{2_{001}\|\frac{1}{2}0\frac{1}{2}\}$
	z,x,y,+1	$\{3^+_{111}\|0\}$
	y,z,x,+1	$\{3^-_{111}\|0\}$
	−y,z+$\frac{1}{2}$,−x+$\frac{1}{2}$,+1	$\{3^-_{1-1-1}\|0\frac{1}{2}\frac{1}{2}\}$
	−z+$\frac{1}{2}$,−x,y+$\frac{1}{2}$,+1	$\{3^+_{1-1-1}\|\frac{1}{2}0\frac{1}{2}\}$
	−y+$\frac{1}{2}$,−z,x+$\frac{1}{2}$,+1	$\{3^-_{-11-1}\|\frac{1}{2}0\frac{1}{2}\}$
	z+$\frac{1}{2}$,−x+$\frac{1}{2}$,−y,+1	$\{3^+_{-11-1}\|\frac{1}{2}\frac{1}{2}0\}$
	y+$\frac{1}{2}$,−z+$\frac{1}{2}$,−x,+1	$\{3^-_{-1-11}\|\frac{1}{2}\frac{1}{2}0\}$
	−z,x+$\frac{1}{2}$,−y+$\frac{1}{2}$,+1	$\{3^+_{-1-11}\|0\frac{1}{2}\frac{1}{2}\}$
MSG symmetry centering operations	x,y,z,+1	$\{1\|0,0,0\}$
Magnetic symmetry constraints	m_x, m_x, m_x	

AOM CALCULATIONS

Graphical schemes provide an overview of low-energy states for Fe^{II} ions in ligand fields of D_{3d} and C_{3v} symmetry including spin-orbit coupling, Fig. S5. Fig. S6 illustrates the field dependence of the lowest energy levels. The calculated temperature-dependent magnetic susceptibility for both ligand fields is provided in Fig. S7, which focuses on the single-ion anisotropy in the purely paramagnetic state.

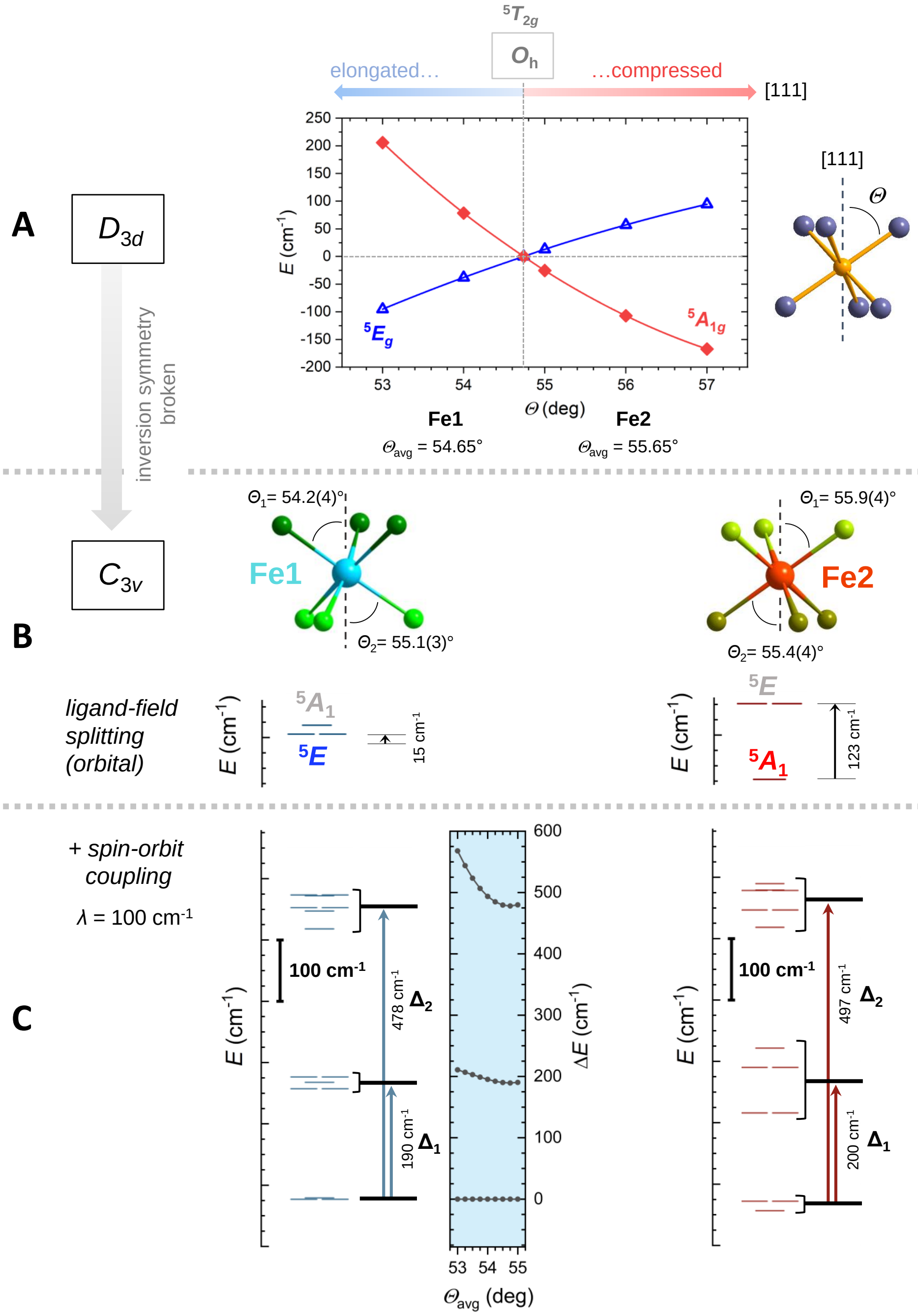

FIG. S5. AOM calculations based on the room temperature crystal structure. (A) Splitting of the $^5T_{2g}$ ground state (O_h symmetry) under trigonal distortion (elongation/compression) of the $[FeO_6]$ polyhedron along the three-fold axis (D_{3d}). (B) Displacement of Fe-ions along the [111] axis breaks inversion symmetry (C_{3v} symmetry). (C) Energy level diagrams including spin-orbit coupling. We grouped the calculated energies into three levels with averaged energies ($\Delta_{1,2}$) referring to the fits of $QS(T)$, see main text. The blue shaded graph illustrates the angular dependence of $\Delta_{1,2}$ for the degenerate case (Fe1).

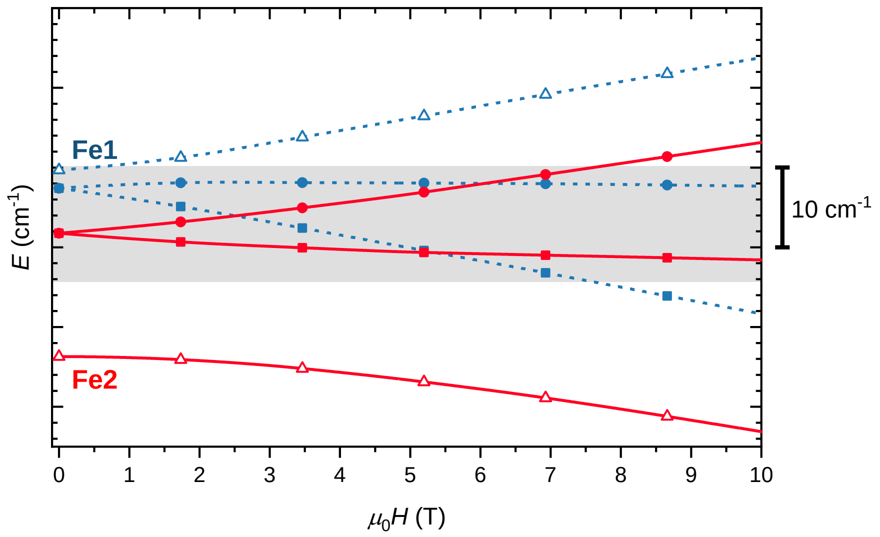

FIG. S6. Zeeman splitting of the three lowest spin-orbit coupled energy levels of each Fe site obtained from the AOM calculations. Note the similar energies of the magnetically active levels (doubly degenerate levels in zero field – filled symbols) emphasized by the shaded area.

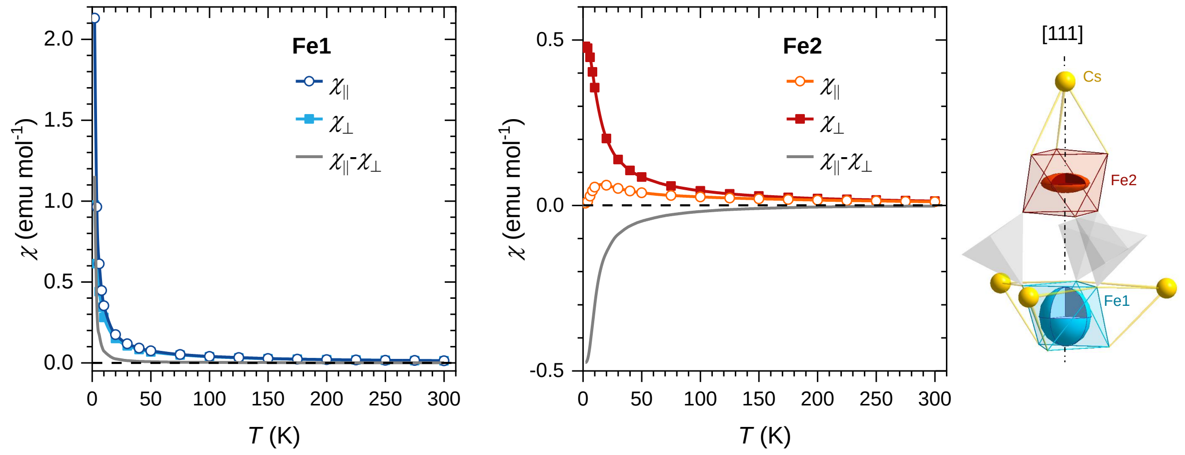

FIG. S7. Calculated (AOM) magnetic susceptibility of paramagnetic Fe-sites in $Cs_2Fe_2(MoO_4)_3$ with $\chi_\parallel$ along the $\langle 111 \rangle$ axis. The anisotropy is shown in gray as $\chi_\parallel - \chi_\perp$. Easy axis for Fe1 and easy plane anisotropy for Fe2. (Right) Single-ion anisotropies of the individual sites within the *nn* dimer. Ellipsoids represent the calculated direction and magnitude of the Fe^{II} magnetic moments at 4 K.

DIPOLE-DIPOLE INTERACTION

An order-of-magnitude estimate of the magnetic dipole-dipole interaction between the Fe^{II} ions in the paramagnetic state is provided based on the AOM calculation and the room-temperature structure in Tab. S5.

We calculate the order-of-magnitude estimate of the energy for magnetic dipole-dipole interaction between the Fe^{II} magnetic moments by the approximation of fixed permanent dipoles with freely rotating dipolar moments. These are thermally averaged over all angles (paramagnetic analog of the Keesom interaction [3]). For the calculation, we used the distances between Fe^{II} ions from the room-temperature structure data (Tab. 1 in the main text) and the effective magnetic moments obtained from AOM calculations (Tab. S5) set into the following equation:

$$\overline{w}_{dd,\mathrm{A}}(T, r_{\mathrm{A}i}) = -\sum_{i=1}^{13} \frac{2}{3} \frac{1}{k_{\mathrm{B}}T} \left(\frac{\mu_0}{4\pi} \frac{\mu_{\mathrm{A}}\mu_i}{r_{\mathrm{A}i}^3} \right)^2 . \tag{S1}$$

Here, $\overline{w}_{dd,\mathrm{A}}$ denotes the energy of the dipole-dipole interaction of Fe^{II} at the selected site (A = Fe1 or Fe2), with the effective magnetic moment μ_{A}, and other Fe_i^{II} ions ("B-site") at a distance $r_{\mathrm{A}i}$, which have the effective magnetic moment of μ_i. The sum runs over all nearest and next-nearest Fe^{II} ions up to a distance of 7 Å (in total 13 neighbors); μ_0 is the permeability of vacuum. The overview of calculated energy $\overline{w}_{dd,\mathrm{A}}$ for individual Fe sites and selected temperatures is provided in Tab. S5.

While this approximation is more precise at higher temperatures, at which the system is in a pure paramagnetic regime with diminished spin-orbit-coupling anisotropy, it still provides a reasonable estimate of the energy scale. Importantly, in the static case, the magnetic dipole-dipole interaction between the nearest neighbors practically vanishes due to their different single-ion anisotropies, which would render their magnetic moments approximately perpendicular.

TABLE S5. Effective magnetic moments of Fe^{II} ions in Fe1 and Fe2 sites obtained from AOM calculations, and the calculated effective magnetic dipole-dipole interaction according to Eq. S1 at selected temperatures.

A-site	T(K)	$\mu_{\mathrm{eff}}(\mu_{\mathrm{B}})$	$\overline{w}_{dd,\mathrm{A}}$(μeV)	$\overline{w}_{dd,\mathrm{A}}$(cm^{-1})	$\overline{w}_{dd,\mathrm{A}}$(mK)	A-site	T(K)	$\mu_{\mathrm{eff}}(\mu_{\mathrm{B}})$	$\overline{w}_{dd,\mathrm{A}}$(μeV)	$\overline{w}_{dd,\mathrm{A}}$(cm^{-1})	$\overline{w}_{dd,\mathrm{A}}$(mK)
Fe1	2	4.664	0.38	$3.1\cdot10^{-3}$	4.6	Fe2	2	2.272	0.23	$1.9\cdot10^{-3}$	2.7
	4	4.832	0.33	$2.7\cdot10^{-3}$	3.8		4	3.204	0.27	$2.2\cdot10^{-3}$	3.1
	300	5.515	0.014	$1.2\cdot10^{-3}$	0.17		300	5.513	0.016	$1.3\cdot10^{-3}$	0.19

^{57}FE MÖSSBAUER SPECTROSCOPY

Representative spectra at 4 K and 250 K are shown in Fig. S8. The temperature dependence of line intensities and center shift are plotted in Fig. S9. Hyperfine parameters for ^{57}Fe Mössbauer spectra of $Cs_2Fe_2(MoO_4)_3$ are given in Tab. S6.

The transmission Mössbauer spectra of $Cs_2Fe_2(MoO_4)_3$ were analyzed with the Recoil software [4] by using the Extended Voigt-based Fitting (xVBF) analysis [5] to extract the Gaussian distribution of the quadrupole splitting and hyperfine magnetic field for each site. Representative fits of the ^{57}Fe Mössbauer spectra for the lowest and highest measured temperatures are shown in Fig. S8 and the parameters obtained from the fits are summarized in Tab. S6. The full width at half maximum of the Lorentzian natural line was fixed to 0.28 mm s^{-1}. For temperatures below 22 K, the Fe1 subspectrum was fitted by two independent components with a fixed center shift, δ_{exp}, to obtain the information on the fluctuating hyperfine field, H_{hf}, as well as quadrupole splitting, QS. At higher temperatures, both Fe1a and Fe1b subspectra were linked as subcomponents of a quadrupole-split component with identical δ_{exp}. The provided errors of hyperfine parameters obtained from the fits were calculated by using Monte Carlo sampling with counting statistics and correspond to the 95% confidence interval. The absence of any discontinuity in the hyperfine parameters of Fe1 and Fe2 subspectra excludes structural phase transitions.

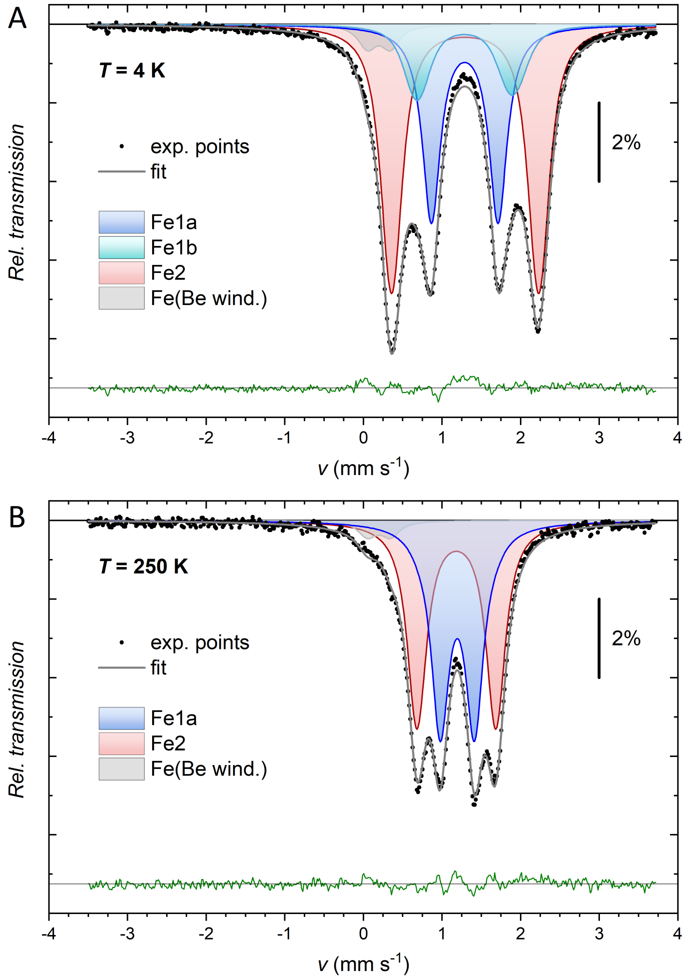

FIG. S8. Transmission ^{57}Fe Mössbauer spectra of $Cs_2Fe_2(MoO_4)_3$ at (A) 4 K and (B) 250 K showing the individual spectral components. The bottom green line gives the difference between the fit and experimental data.

The intensity of a spectral component I is directly proportional to the Lamb-Mössbauer (recoil-free) factor f of the respective site, whose temperature dependence also determines $I(T)$. Similarly to Debye-Waller factor in XRD, the Lamb-Mössbauer factor is related to the mean square displacement of the nucleus from its equilibrium position, $\langle x^2 \rangle$, in the direction of propagation of γ-rays [6]. The intensities of individual spectral components were calculated from

the spectral area A normalized by the respective background intensity I_{bg} as $I = A/I_{\mathrm{bg}}$ and are depicted in Fig. S9A.

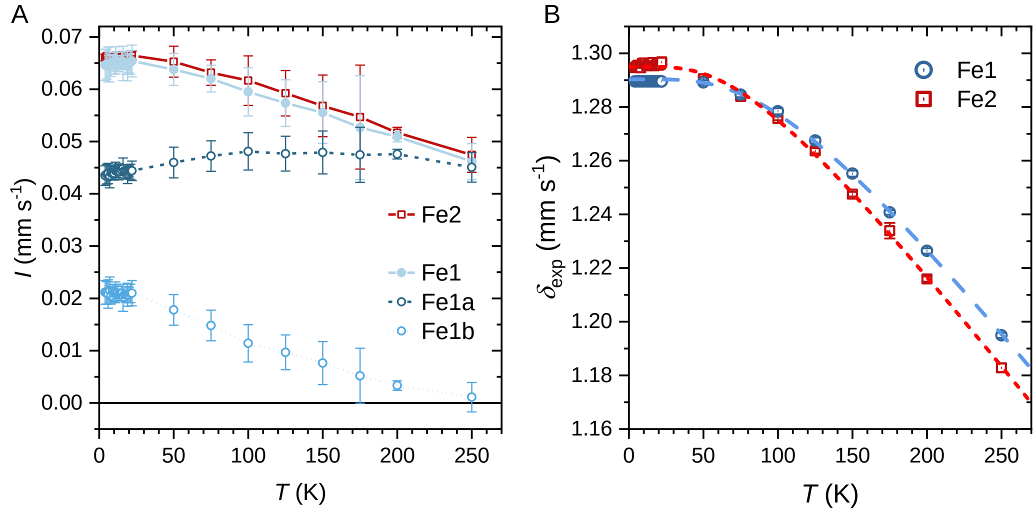

FIG. S9. (A) Intensity of individual components in the ^{57}Fe Mössbauer spectra of $Cs_2Fe_2(MoO_4)_3$, $I(\mathrm{Fe1}) = I(\mathrm{Fe1a}) + I(\mathrm{Fe1b})$. (B) Temperature dependence of the center shift for both Fe-sites in $Cs_2Fe_2(MoO_4)_3$, $\delta_{\mathrm{exp}}(\mathrm{Fe1a}) = \delta_{\mathrm{exp}}(\mathrm{Fe1b})$. Fits in the temperature range of 22–250 K according to Eq. S2 are shown as dashed lines.

The center shift is composed of two contributions, the isomer shift, δ_0, which results from the electric monopole interaction of the ^{57}Fe nucleus and its surroundings, and the second-order Doppler shift, δ_{SOD}. The δ_{SOD} shift originates in a relativistic change in energy of the γ-photon due to thermal vibrations of the ^{57}Fe nuclei, and is proportional to the mean square velocity $\langle v^2 \rangle$ (or mean kinetic energy) of the ^{57}Fe atom. From the temperature dependence of δ_{SOD}, we determine the Mössbauer temperature, θ_{M}, an analog of the Debye temperature specific for the Fe nuclei in their environment. The fits of $\delta_{\mathrm{exp}}(T)$ to Eq. S2 [6, 7] are depicted in Fig. S9B.

$$\delta_{\mathrm{exp}}(T) = \delta_0 + \delta_{\mathrm{SOD}}(T) = \delta_0 - \frac{9k_{\mathrm{B}}E_\gamma}{16M_{\mathrm{eff}}c^2}\left(\theta_{\mathrm{M}} + 8T\left(\frac{T}{\theta_{\mathrm{M}}}\right)^3 \int_0^{\theta_{\mathrm{M}}/T} \frac{x^3}{e^x - 1}\mathrm{d}x\right), \tag{S2}$$

where $E_\gamma = 14.41\,\mathrm{keV}$ is the energy of the Mössbauer transition, M_{eff} is the effective mass of the vibrating ^{57}Fe nuclei (fixed to the isotopic mass of ^{57}Fe, 53095 MeV c^{-2}, to stabilize the fit).

We obtain $\delta_0(\mathrm{Fe1}) = 1.40\,\mathrm{mm\,s^{-1}}$, $\theta_{\mathrm{M}}(\mathrm{Fe1}) = 404(4)\,\mathrm{K}$ and $\delta_0(\mathrm{Fe2}) = 1.38\,\mathrm{mm\,s^{-1}}$, $\theta_{\mathrm{M}}(\mathrm{Fe2}) = 308(8)\,\mathrm{K}$. The θ_{M} values evidence the disparate ground states of the two Fe-sites.

TABLE S6. Parameters obtained from the xVBF fit of ^{57}Fe Mössbauer spectra of $Cs_2Fe_2(MoO_4)_3$. For each component, δ_{exp} denotes the center (isomer) shift, QS and σ_{QS} the average and the Gaussian width of the quadrupole splitting distribution, H_{hf} the effective magnetic hyperfine field, A_{rel} the relative ratio of the site in $Cs_2Fe_2(MoO_4)_3$ (excluding Fe in the Be window) obtained from the spectral area A, and I is the spectral intensity. The change in models at 22 K used for the Fe1 site is marked by the horizontal line.

	Fe1a						Fe1b						Fe2					
T (K)	δ_{exp} (mm s^{-1})	QS (mm s^{-1})	σ_{QS} (mm s^{-1})	H_{hf}‡ (kOe)	A_{rel} (%)	I (mm s^{-1})	δ_{exp} (mm s^{-1})	QS (mm s^{-1})	σ_{QS} (mm s^{-1})	H_{hf} (kOe)	A_{rel} (%)	I (mm s^{-1})	δ_{exp} (mm s^{-1})	QS (mm s^{-1})	σ_{QS} (mm s^{-1})	H_{hf} (kOe)	A_{rel} (%)	I (mm s^{-1})
4	1.2895*	0.849(3)	0.00(2)	0.1(4)	33(1)	0.043(2)	1.2895*	1.21(2)	0.16(4)	4.0(4)	16(1)	0.021(2)	1.2947(4)	1.871(2)	0.098(3)	0.0(7)	51(1)	0.0661(6)
5	1.2895*	0.839(4)	0.02(1)	0.1(5)	33.3(9)	0.044(2)	1.2895*	1.21(2)	0.18(4)	2.3(5)	16(1)	0.021(2)	1.2950(4)	1.869(2)	0.103(4)	0.0(7)	51(1)	0.0664(4)
6	1.2895*	0.834(4)	0.03(2)	0.0(8)	34(1)	0.044(2)	1.2895*	1.22(2)	0.18(5)	3(1)	16(2)	0.021(3)	1.2955(7)	1.866(3)	0.100(5)	0.0(6)	50(1)	0.065(1)
7	1.2895*	0.822(4)	0.043(6)	0.1(6)	33(1)	0.043(2)	1.2895*	1.19(2)	0.19(5)	2.8(6)	16(1)	0.021(3)	1.2951(5)	1.861(3)	0.106(3)	0.0(9)	50(1)	0.0658(7)
8	1.2895*	0.813(4)	0.00(2)	0.8(6)	33.7(7)	0.044(1)	1.2895*	1.20(2)	0.18(3)	2(1)	15.7(9)	0.020(2)	1.2946(7)	1.855(1)	0.106(4)	0.0(7)	50.6(8)	0.0660(4)
9	1.2895*	0.806(3)	0.01(2)	0.4(4)	33.4(5)	0.044(1)	1.2895*	1.20(1)	0.20(2)	3.1(7)	16.2(6)	0.021(1)	1.2962(6)	1.854(2)	0.100(3)	0.0(8)	50.3(5)	0.0661(2)
10	1.2895*	0.798(3)	0.01(1)	0.0(5)	33.6(7)	0.044(1)	1.2895*	1.19(2)	0.18(3)	3.3(5)	16.1(9)	0.021(2)	1.2964(4)	1.848(2)	0.100(3)	0.0(6)	50.4(8)	0.0659(4)
11	1.2895*	0.791(4)	0.00(2)	0.9(6)	33.7(9)	0.044(2)	1.2895*	1.19(3)	0.20(5)	3(1)	16(1)	0.021(2)	1.2964(7)	1.847(2)	0.100(4)	0.0(8)	50.2(9)	0.0662(7)
12	1.2895*	0.783(3)	0.03(3)	0.0(9)	33.7(7)	0.044(1)	1.2895*	1.19(3)	0.20(3)	3(1)	15.9(8)	0.021(1)	1.2954(9)	1.840(4)	0.100(7)	0.4(6)	50.3(7)	0.066(1)
13	1.2895*	0.772(5)	0.01(2)	0.8(6)	33.6(8)	0.044(1)	1.2895*	1.18(2)	0.16(3)	3(1)	16(1)	0.0207(1)	1.2958(9)	1.834(2)	0.105(3)	0.0(8)	50.6(9)	0.0664(5)
14	1.2895*	0.771(2)	0.00(2)	0.2(5)	33.7(4)	0.0442(6)	1.2895*	1.190(7)	0.19(2)	3(1)	15.9(5)	0.021(1)	1.2957(7)	1.833(2)	0.102(3)	0.0(7)	50.3(4)	0.0659(4)
15	1.2895*	0.762(1)	0.03(1)	0.2(3)	33.8(6)	0.045(1)	1.2895*	1.18(2)	0.19(2)	3.5(7)	15.9(7)	0.021(1)	1.2962(3)	1.829(2)	0.109(3)	0.0(6)	50.3(6)	0.0662(6)
16	1.2895*	0.759(4)	0.03(1)	0.0(7)	34(1)	0.045(2)	1.2895*	1.18(3)	0.20(4)	3(1)	15(1)	0.020(3)	1.2965(5)	1.824(2)	0.109(5)	0.2(7)	50(1)	0.0659(8)
17	1.2895*	0.754(2)	0.02(2)	0.2(7)	33.8(1)	0.0444(2)	1.2895*	1.161(6)	0.192(9)	3.4(5)	15.8(1)	0.0207(2)	1.2954(8)	1.821(1)	0.106(3)	0.0(7)	50.4(1)	0.0662(3)
18	1.2895*	0.748(3)	0.00(2)	0.6(6)	33.6(6)	0.044(1)	1.2895*	1.16(1)	0.20(3)	3.3(8)	15.9(8)	0.021(1)	1.2955(8)	1.817(2)	0.110(4)	0.4(8)	50.5(7)	0.0659(7)
19	1.2895*	0.745(4)	0.02(2)	0.0(8)	33.5(9)	0.044(2)	1.2895*	1.16(3)	0.18(4)	4.1(6)	16(1)	0.021(2)	1.2962(7)	1.815(3)	0.105(6)	1.0(8)	51(1)	0.0662(8)
20	1.2895*	0.740(2)	0.03(1)	0.3(5)	33.8(6)	0.044(1)	1.2895*	1.16(1)	0.21(3)	3.7(6)	15.8(8)	0.021(2)	1.2956(7)	1.813(2)	0.110(2)	0.0(7)	50.4(7)	0.0660(3)
21	1.2895*	0.737(3)	0.00(2)	0.0(7)	33.7(8)	0.044(1)	1.2895*	1.16(2)	0.23(3)	1(2)	16.0(9)	0.021(2)	1.2958(8)	1.808(2)	0.096(3)	0.8(6)	50.3(8)	0.0660(4)
22	1.2895(9)	0.732(6)	0.00(3)	-	35(2)	0.044(2)	1.2895(9)	1.17(4)	0.21(6)	-	15(2)	0.021(2)	1.2968(8)	1.806(3)	0.099(6)	-	50.4(4)	0.0665(8)
50	1.2891(9)	0.673(3)	0.00(2)	-	36(2)	0.046(3)	1.2891(9)	1.18(6)	0.25(8)	-	14(2)	0.018(3)	1.2905(8)	1.750(5)	0.103(6)	-	51(1)	0.065(3)
75	1.2846(9)	0.647(3)	0.00(2)	-	38(2)	0.047(3)	1.2846(9)	1.19(5)	0.23(9)	-	12(2)	0.015(3)	1.284(1)	1.673(5)	0.083(6)	-	50(1)	0.063(2)
100	1.278(1)	0.616(3)	0.00(2)	-	40(3)	0.048(4)	1.278(1)	1.20(8)	0.16(9)	-	9(3)	0.011(4)	1.2757(7)	1.580(6)	0.07(2)	-	51(2)	0.062(5)
125	1.268(1)	0.587(3)	0.00(2)	-	41(3)	0.048(3)	1.268(1)	1.16(8)	0.2(1)	-	8(3)	0.010(3)	1.264(1)	1.479(9)	0.08(1)	-	51(2)	0.059(4)
150	1.255(1)	0.548(3)	0.00(2)	-	43(4)	0.048(4)	1.255(1)	1.14(8)	0.2(1)	-	7(4)	0.008(4)	1.2475(8)	1.37(1)	0.08(1)	-	51(3)	0.057(6)
175	1.2407(9)	0.516(2)	0.00(2)	-	44(5)	0.047(5)	1.2407(9)	1.1(1)	0.2(2)	-	5(5)	0.005(5)	1.234(3)	1.258(9)	0.08(2)	-	51(5)	0.06(1)
200	1.226(1)	0.486(2)	0.00(2)	-	46.4(9)	0.0476(9)	1.226(1)	1.2(2)	0.0(1)	-	3.2(9)	0.0033(9)	1.216(1)	1.156(5)	0.079(8)	-	50.4(5)	0.052(1)
250	1.195(1)	0.436(3)	0.00(2)	-	48(3)	0.045(3)	1.195(1)	1.0(1)	0*	-	1(3)	0.001(3)	1.183(2)	1.003(7)	0.054(9)	-	51(2)	0.047(3)

*Fixed values

‡Average hyperfine field of ≈0.3 kOe.

HEAT CAPACITY DATA

Details on the fitting procedure of the heat capacity by the Debye-Einstein integral to extract the magnetic heat capacity are described, the parameters are listed in Tab. S7. The fitted curve of the phonon contribution along with the experimental data and an isotypic non-magnetic compound $Cs_2Mg_2(MoO_4)_3$ is illustrated in Fig. S10. The extracted magnetic heat capacity C_m/R of $Cs_2Fe_2(MoO_4)_3$ and its low-temperature fits are presented in Fig. S11. The simulations of magnetic heat capacity corresponding to Schottky anomalies based on lowest energy levels of the two Fe sites and the corresponding magnetic entropy change is compared with experimental values in Fig. S12 for different applied magnetic fields.

The experimental heat capacity measured at zero field was fitted by the Debye-Einstein integral to approximate the phonon contribution:

$$\frac{C_\mathrm{p}}{T} = \frac{1}{T}\left[mD(\theta_\mathrm{D}/T) + \sum_{i=1}^{j} n_i E_i(\theta_{\mathrm{E},i}/T)\right], \tag{S3}$$

in which the Debye integral is defined as

$$D(\theta_\mathrm{D}/T) = 3R\left(\frac{T}{\theta_\mathrm{D}}\right)^3 \int_0^{\theta_\mathrm{D}/T} \frac{x^4 e^x}{(e^x-1)^2}\mathrm{d}x, \tag{S4}$$

and the Einstein terms by

$$E(y) = R\frac{y^2 e^y}{(e^y-1)^2}, \text{ where } y = \frac{\theta_\mathrm{E}}{T}. \tag{S5}$$

In these equations, θ_D and θ_E denote the Debye and Einstein temperatures, R the gas constant, m and n_i are fitting parameters approximating the number of the respective modes, whose sum $m+\sum_i n_i$ is equal three-times the number of atoms in the formula unit (i.e., 57 in our case). In the fitting procedure, all three acoustic – Debye – modes are approximated by a single Debye temperature (θ_{D_1}, D_1 group with $m = 3$). The remaining optical modes are clustered into four groups ($j = 4$) described by four Einstein temperatures (θ_{E_i}). To reduce the number of free parameters, the anharmonic corrections (see, e.g., [8, 9]) were not applied.

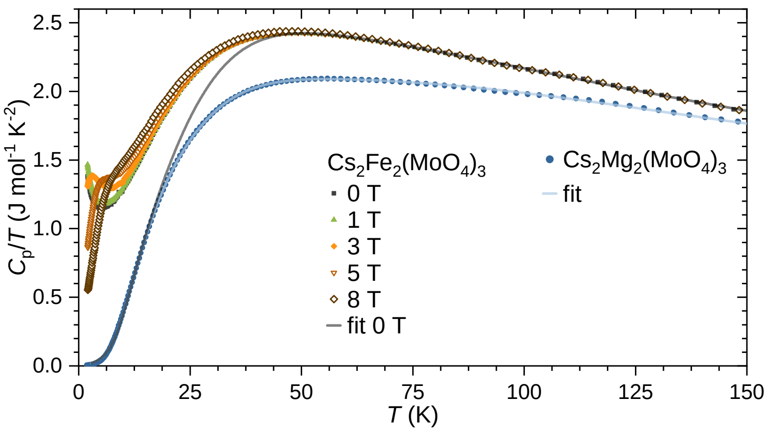

FIG. S10. Molar heat capacity of $Cs_2Fe_2(MoO_4)_3$, C_p/T is shown per formula unit in various applied fields, along with the isotypic Mg-compound data for comparison. Fits are represented as lines.

For consistency, the fitting parameters were compared to other Cs-containing molybdates, such as $Cs_2Mo_2O_7$ [10] and $Cs_2Mo_2O_7 \cdot CsX$ (X = Cl, I) [11]. These provide an estimate for the high-energy Einstein modes, which correspond primarily to fundamental stretching vibrations (E_4) and bending modes (E_3) of the molybdate, while E_1 is dominated by the heavy Cs^+ counterions. We used the diamagnetic isotypic $Cs_2Mg_2(MoO_4)_3$ compound [12] to derive the Debye temperature (θ_{D_1}) from a fit of C_p/T vs. T^2 plot at low temperatures. The model parameters obtained from fits to the Debye-Einstein integral for this compound are provided in Tab. S7.

TABLE S7. Model parameters – Debye and Einstein temperatures and numbers of the respective modes – used for fitting zero-field heat capacity by Eq. S3 between 50 and 150 K for $Cs_2Fe_2(MoO_4)_3$ and $Cs_2Mg_2(MoO_4)_3$ (fitted range 3–280 K). Fixed parameters are marked by the asterisk, the errors provided are a rough estimate.

	$Cs_2Fe_2(MoO_4)_3$		$Cs_2Mg_2(MoO_4)_3$	
	$\theta_{D,E}$ (K)	m,n	$\theta_{D,E}$ (K)	m,n
D_1	96*	3*	96(2)	3*
E_1	71*	4.6*	71(2)	4.7(2)
E_2	151(3)	15.8(4)	160(4)	12.5(2)
E_3	405(7)	19.0(2)	400(5)	22.3(2)
E_4	993*	14.6(3)	993(10)	14.5(2)

A simple subtraction of neither raw nor re-scaled $C_p(T)/T$ data for the Mg-compound provide a reasonable estimate of the magnetic heat capacity of $Cs_2Fe_2(MoO_4)_3$, see Fig. S10. Mg^{II} and Fe^{II} differ (i) in atomic weight, (ii) with respect to their ionic radii in six-fold coordination (0.72 Å for Mg^{II} and 0.78 Å for high-spin Fe^{II}, [13]), and (iii) in terms of bonding (ionic vs. coordinate). In order to model the phonon part of the specific heat for $Cs_2Fe_2(MoO_4)_3$, we fixed θ_{D_1}, θ_{E_1}, θ_{E_4} and the corresponding numbers of modes to the respective values of the Mg-compound. Then we fitted parameters of E_2 and E_3 modes, in which the vibrations within the $[FeO_6]$ polyhedra are dominantly involved (see also Mössbauer temperatures θ_M for the two iron sites). Note that while the Einstein temperatures do not differ significantly, the redistribution of mode numbers indicates the differences in bonding for the $[FeO_6]$ complex. The final fitting parameters obtained for $Cs_2Fe_2(MoO_4)_3$ in zero applied field are summarized in Tab. S7 and were used to apply the phonon correction for $C_p(T)$ to extract the magnetic contribution, $C_m(T)$.

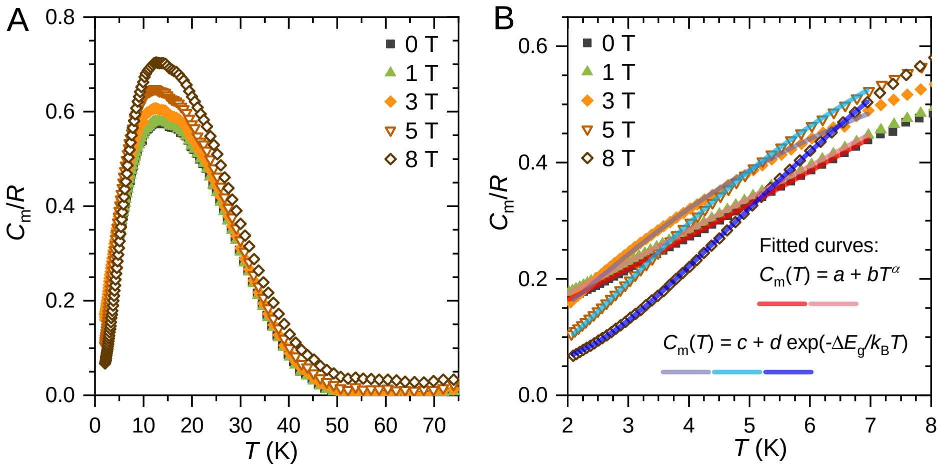

FIG. S11. Magnetic heat capacity, obtained by subtraction of the fitted phonon contribution (A) and low-temperature fits (B) provided per mol Fe.

TABLE S8. Parameters obtained from the fit of magnetic heat capacity $C_m(T)/R$ of $Cs_2Fe_2(MoO_4)_3$ in various applied magnetic fields, the corresponding equations are shown in the Fig. S11 B. The model indicated in black was preferred for the respective field based on the corrected R^2 and reasonable parameters, e.g. in the case of 3 T.

	$C_m(T)/R = a + b\,T^\alpha$				$C_m(T)/R = c + d\exp(-\Delta E_g/k_BT)$			
μ_0H (T)	a	b ($K^{-\alpha}$)	α	$R^2_{corr.}$	c	d	ΔE_g (cm^{-1})	$R^2_{corr.}$
0	0.066(2)	0.0466(9)	1.074(8)	0.99993	0.151(1)	0.89(1)	5.53(8)	0.99933
1	0.070(1)	0.0518(7)	1.024(6)	0.99996	0.161(2)	0.87(1)	5.43(8)	0.9992
3	-0.36(2)	0.39(2)	0.40(1)	0.99986	0.093(4)	0.811(6)	3.53(7)	0.99931
5	-0.31(3)	0.26(2)	0.60(3)	0.9991	0.0411(6)	1.153(2)	4.20(1)	0.99998
8	-0.063(8)	0.048(4)	1.28(3)	0.99889	0.0493(6)	1.713(8)	6.40(3)	0.99994

The magnetic heat capacity $C_m(T)$ was fitted in the temperature range of 2–7 K by a power law and a model

simulating an energy gap of ΔE_g, see Fig. S11B and Tab. S8. At zero applied field and at 1 T, the power law provided exponents of $\alpha(0\,\mathrm{T}) = 1.074(8)$ and $\alpha(1\,\mathrm{T}) = 1.024(6)$. Data obtained in applied magnetic fields of 3 T could be perceived at the transition between the two models. For higher fields, the data are described by the exponential fit, providing the energy gap of ΔE_g.

Next we compare experimental $C_m/T(T)$ data in applied magnetic fields with the calculated magnetic heat capacity based on a three-level Schottky model [14, 15]:

$$\frac{C_\mathrm{m}(T)}{R} = \frac{1}{Z^2(k_\mathrm{B}T)^2}\left[Z\sum_{i=0}^{2} E_i^2 e^{-\frac{E_i}{k_\mathrm{B}T}} - \left(\sum_{i=0}^{2} E_i e^{-\frac{E_i}{k_\mathrm{B}T}}\right)^2\right], \quad \text{where} \quad Z = \sum_{i=0}^{2} e^{-\frac{E_i}{k_\mathrm{B}T}}. \tag{S6}$$

Here, E_i refer to the Fe-site specific energies (given in Fig. S6) and Z is the partition function. We show the averaged value, $C_m(T)/T$, over the two Fe-sites in Fig S12. We conclude that the magnetic contribution to the experimental heat capacity is not explained merely by Schottky-like anomalies of non-interacting (purely paramagnetic) Fe^{II}-ions.

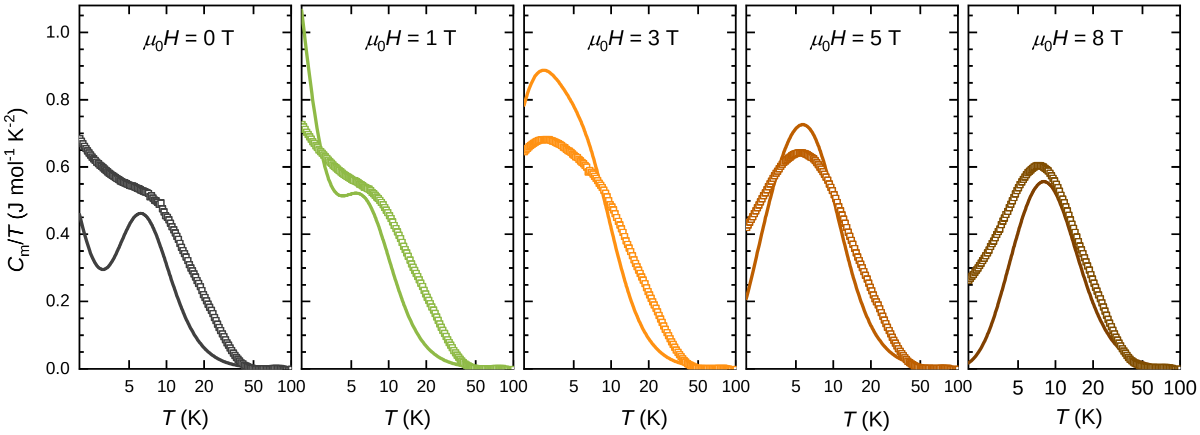

FIG. S12. Experimental (empty symbols) and calculated (lines) magnetic heat capacity C_m/T. Calculations are based on a three-level Schottky model (Eq. S6) for each Fe-site and then averaged to conform with C_m/T in units per mol Fe.

MAGNETIZATION DATA

Comparison of DC magnetic susceptibility measured in zero-field-cooled and field-cooled modes in selected fields and the reciprocal susceptibility with Curie-Weiss fits are presented in Fig. S13. We used $\chi^{-1} = [C/(T - \theta_{\mathrm{CW}}) + \chi_{\mathrm{TIP}}]^{-1}$, where temperature-independent paramagnetism was fitted at $\mu_0 H = 10\,\mathrm{mT}$ ($\chi_{\mathrm{TIP}} \approx 7{\cdot}10^{-3}\,\mathrm{emu\,mol^{-1}}$) and 100 mT ($\chi_{\mathrm{TIP}} \approx 2{\cdot}10^{-3}\,\mathrm{emu\,mol^{-1}}$) for a fixed $\theta_{\mathrm{CW}} = -22\,\mathrm{K}$.

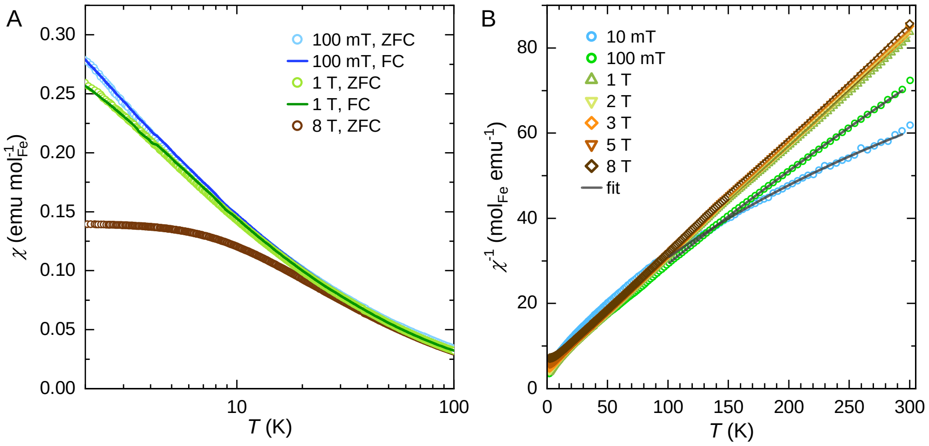

FIG. S13. (A) Comparison of the DC susceptibility $\chi(T)$ per mol Fe of $Cs_2Fe_2(MoO_4)_3$ measured in the zero-field-cooled (ZFC) and field-cooled (FC) modes. (B) Reciprocal DC susceptibility (ZFC mode), the lines represent respective Curie-Weiss fits.

At high temperatures the effective exchange can be approximated by a mean field approach (S7):

$$\theta_{\mathrm{CW}} = \frac{1}{3} \cdot S(S+1) \cdot (J_1 + 3J_2 + 3J_3 + 6J_4 + 6J_5), \tag{S7}$$

with the multiplicity given in Tab.1 (main text). Using $S = 2$ for both Fe-sites gives J_{eff} = -11 K (AFM). This is reasonably close to the temperature adjustment given for the simulation of the magnetization data at 100 K (Fig. S14 left panel). At 10 K Fe1 ($S = 2$) and Fe2 ($S' = 1$) holds for which we give averaged calculated magnetization (blue dashed lines) based on the respective Brillouin functions. Simulation of the experimental data at selected temperatures are provided as dotted lines with T_2 referring to the adjusted Brillouin function for $S = 2$ and T_1 in reference to $S = 1$ (Fig. S14 middle and right panel).

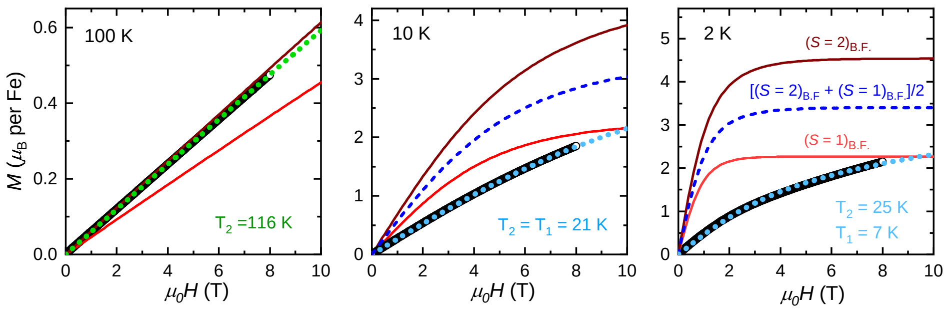

FIG. S14. Experimental field dependent magnetization data per Fe (black circles) for $Cs_2Fe_2(MoO_4)_3$ at selected temperatures. Calculated magnetization curves ($g_{\mathrm{eff}} = 2.27$) using Brillouin functions (B.F.) for paramagnetic $S = 2$ (brown line), $S = 1$ (red line), and the mixed spin state scenario (dashed blue line) are given for reference at experimental temperatures. Simulations (dotted lines) refer to the sum of the magnetization derived from the respective B.F.'s for $S = 2$ and $S = 1$ at temperatures T_2 and T_1, respectively.

The calculation of magnetocaloric parameters is based on the field-dependent magnetization curves measured at various temperatures (Fig. S15).

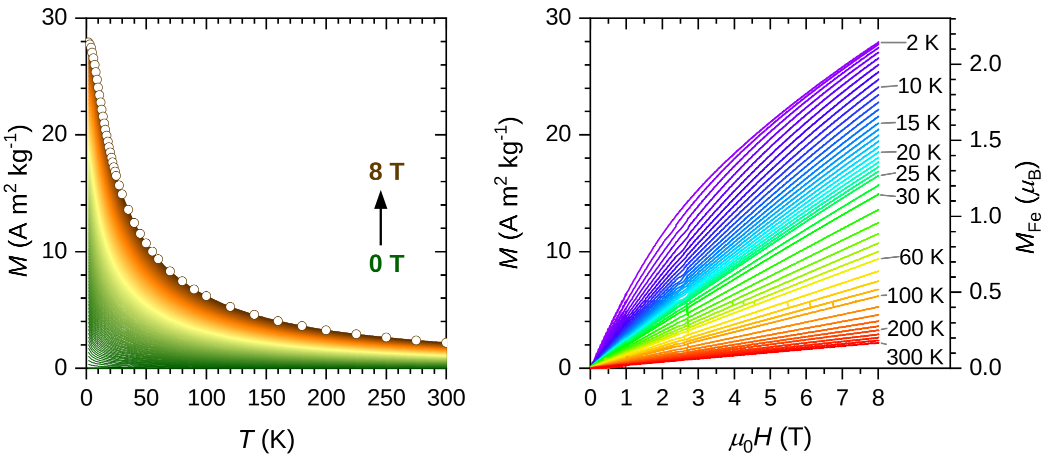

FIG. S15. Specific magnetization of $Cs_2Fe_2(MoO_4)_3$, employed in the calculation of magnetocaloric parameters. Temperature-dependent magnetization at various fields between 0 and 8 T (left panel), derived from the curves shown in the right panel (lines at 0.05 T increments). Field-dependent scans measured at various temperatures; the ripples in the measured data around $M \approxeq 5\,\mathrm{A\,m^2\,kg^{-1}}$ and $\mu_0 H \approxeq 2.75\,\mathrm{T}$ are of instrumental origin and relate to change-of-sensitivity and touchdown-centering procedures, respectively.

MAGNETOCALORIC EFFECT

Relevant to the magnetocaloric characteristics of a material are the magnetic entropy change $-\Delta S_\mathrm{m}(T)$ and the adiabatic temperature change ΔT_ad, which are displayed in Fig. S16 for zero initial field and various final magnetic fields.

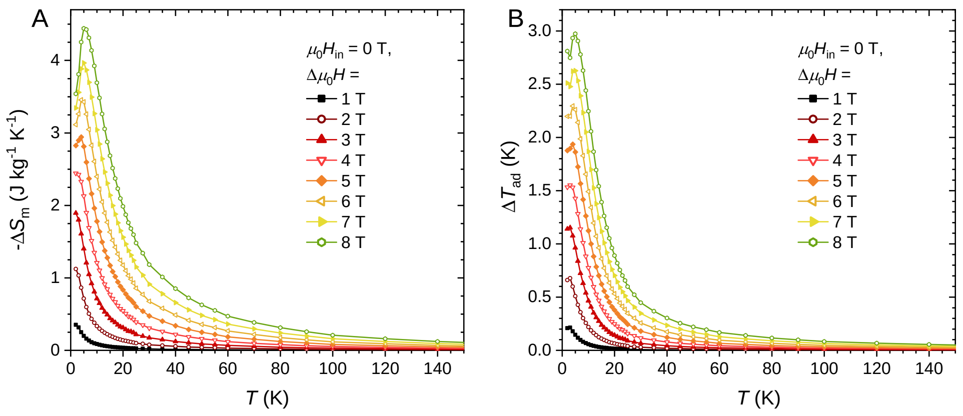

FIG. S16. Magnetocaloric parameters of $Cs_2Fe_2(MoO_4)_3$. (A) The total isothermal magnetic entropy change derived from $M(H)$ isotherms and (B) the total adiabatic temperature change for various differences between the initial and final magnetic fields, with the initial field of 0 T.

The field-dependent magnetic entropy change isotherms were fitted at low- and high-field regions to a power law, whose temperature-dependent exponent n is shown in Fig. S17.

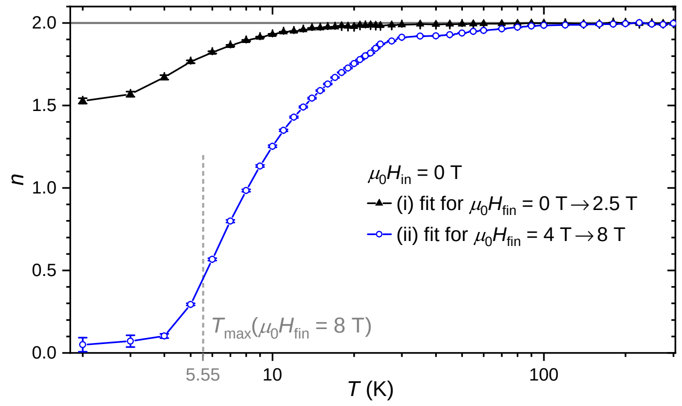

FIG. S17. The exponent n obtained from the fits of field-dependent ΔS_m isotherms by the power law $\Delta S_\mathrm{m}(H,T) = a + b(\mu_0 H)^{n(T)}$ at (i) low magnetic fields in the gapless region and at (ii) high fields in the gapped region (see the main text).

[1] A. A. Coelho, *TOPAS* and *TOPAS-Academic*: an optimization program integrating computer algebra and crystallographic objects written in C++, J. Appl. Cryst. **51**, 210 (2018).

[2] J. Rodríguez-Carvajal, Recent developments for the program FULLPROF, in Commission on Powder Diffraction (IUCr), International Union of Crystallography Newsletter **26**, 12 (2001).

[3] J.-L. Bretonnet, Interactions in atomic and ionic liquids, Advances in Materials **9**, 68 (2020).

[4] K. Lagarec and D. G. Rancourt, Recoil - Mössbauer spectral analysis software for Windows (1998).

[5] K. Lagarec and D. G. Rancourt, Extended Voigt-based analytic lineshape method for determining N-dimensional correlated hyperfine parameter distributions in Mössbauer spectroscopy, Nucl. Instrum. Methods Phys. Res. B: Beam Interact. Mater. At. **129**, 266 (1997).

[6] P. Gütlich, E. Bill, and A. X. Trautwein, *Mössbauer spectroscopy and transition metal chemistry: Fundamentals and applications* (Springer-Verlag, 2011) p. 568.

[7] R. H. Herber, Structure, bonding, and the Mössbauer lattice temperature, in *Chemical Mössbauer Spectroscopy*, edited by R. H. Herber (Springer US, Boston, MA, 1984) pp. 199–216.

[8] C. A. Martín, Simple treatment of anharmonic effects on the specific heat, J. Phys.: Condens.Matter **3**, 5967 (1991).

[9] P. Svoboda, P. Javorský, M. Diviš, V. Sechovský, F. Honda, G. Oomi, and A. A. Menovsky, Importance of anharmonic terms in the analysis of the specific heat of UNi_2Si_2, Phys. Rev. B **63**, 212408 (2001).

[10] A. L. Smith, G. Kauric, L. van Eijck, K. Goubitz, G. Wallez, J.-C. Griveau, E. Colineau, N. Clavier, and R. J. M. Konings, Structural and thermodynamic study of dicesium molybdate $Cs_2Mo_2O_7$: Implications for fast neutron reactors, J. Solid State Chem. **253**, 89 (2017).

[11] A. K. Weber, K. Denisova, P. Lemmens, and A. Möller, Configuration of the dimolybdate in salt inclusion type of compounds, $Cs_2Mo_2O_7{\cdot}CsX$ (X = Cl, Br, and I), Z. anorg. allg. Chem. 10.1002/zaac.202300199 (2023).

[12] $Cs_2Mg_2(MoO_4)_3$ was synthesized by Maurice Beske as a part of his Ph.D. thesis (anticipated publication in 2024) and we obtained his permission to use this isotypic compound as a non-magnetic model system for our analysis of $Cs_2Fe_2(MoO_4)_3$ data.

[13] R. D. Shannon, Revised effective ionic radii and systematic studies of interatomic distances in halides and chalcogenides, Acta Crystallogr., Sect. A: Found. Adv. **32**, 751 (1976).

[14] M. Affronte, J. C. Lasjaunias, A. Cornia, and A. Caneschi, Low-temperature specific heat of Fe_6 and Fe_{10} molecular magnets, Phys. Rev. B **60**, 1161 (1999).

[15] M. Evangelisti, F. Luis, L. J. de Jongh, and M. Affronte, Magnetothermal properties of molecule-based materials, J. Mater. Chem. **16**, 2534 (2006).
